# Genetic alterations in fatty acid transport and metabolism genes are associated with metastatic progression and poor prognosis of human cancers

**DOI:** 10.1038/srep18669

**Published:** 2016-01-04

**Authors:** Aritro Nath, Christina Chan

**Affiliations:** 1 Genetics Program, Michigan State University, East Lansing, Michigan 48824, USA; 2 Department of Chemical Engineering and Materials Science, Michigan State University, East Lansing, Michigan 48824, USA

## Abstract

Reprogramming of cellular metabolism is a hallmark feature of cancer cells. While a distinct set of processes drive metastasis when compared to tumorigenesis, it is yet unclear if genetic alterations in metabolic pathways are associated with metastatic progression of human cancers. Here, we analyzed the mutation, copy number variation and gene expression patterns of a literature-derived model of metabolic genes associated with glycolysis (Warburg effect), fatty acid metabolism (lipogenesis, oxidation, lipolysis, esterification) and fatty acid uptake in >9000 primary or metastatic tumor samples from the multi-cancer TCGA datasets. Our association analysis revealed a uniform pattern of Warburg effect mutations influencing prognosis across all tumor types, while copy number alterations in the electron transport chain gene *SCO2*, fatty acid uptake (*CAV1, CD36*) and lipogenesis (*PPARA, PPARD, MLXIPL*) genes were enriched in metastatic tumors. Using gene expression profiles, we established a gene-signature (*CAV1, CD36, MLXIPL, CPT1C, CYP2E1*) that strongly associated with epithelial-mesenchymal program across multiple cancers. Moreover, stratification of samples based on the copy number or expression profiles of the genes identified in our analysis revealed a significant effect on patient survival rates, thus confirming prominent roles of fatty acid uptake and metabolism in metastatic progression and poor prognosis of human cancers.

Metastases are responsible for the majority of cancer-related mortalities^1^. While the progression of primary malignancies to metastasis may be driven by linear progression of tumors via accumulation of mutations, or by parallel evolution of metastatic tumors, it is evidently clear that the genetic alterations driving tumorigenesis are distinct from the ones involved in metastatic progression^2^,^3^. Cancer cells frequently rewire cellular metabolism to increase ATP synthesis, enhance production of macromolecules and maintain redox status, and the acquisition of such alterations is increasingly being recognized as an important hallmark of cancer^4^,^5^. However, it is unclear if these genetic alterations in metabolic pathways can provide a selective advantage towards the metastatic progression of cancer cells.

One of the most commonly observed, and widely studied, metabolic rewiring event during cellular transformation is the increased uptake of glucose and the switch to aerobic glycolysis (Warburg effect)^6^. Despite the poor efficiency of ATP generation compared to mitochondrial oxidative phosphorylation (OXPHOS), the switch to aerobic glycolysis increases the availability of intermediate metabolites required for anabolic processes including nucleic acid and amino acid biosynthesis that are essential for the sustenance of rapidly proliferating cancer cells^7^. Often ignored, another important source of energy and anabolism in cancer cells is fatty acids (FA) metabolism – almost all tumors gain the ability to synthesize long-chain fatty acids *de novo* by upregulating the expression of fatty acid synthase (*FASN*)^8^. Besides constituting lipid membranes, FAs serve as a rich source of energy production through mitochondrial fatty acid oxidation (FAO). Tumor cells overexpressing the brain isoform of carnitine palmitoyltransferase (*CPT1C*), the enzyme responsible for mitochondrial uptake of FAs, are more aggressive and resistant to therapeutics^9^. From a metabolic perspective, adaptations observed during malignant transformation are very similar in nature to those observed in embryonic and adult hematopoetic stem cells, namely, they metabolize glucose through aerobic glycolysis^10^,^11^. More recently, researchers have established that FA metabolism also plays a major role in the maintenance of stem cells. *De novo* lipogenesis are highly active in adult neural stem cells, and increased *FASN* expression is essential for the proliferation and maintenance of the undifferentiated state of the stem cells^12^. In addition, PPAR-δ (*PPARD*)*-*regulated mitochondrial FA oxidation are critical for the maintenance of hematopoietic stem cells^13^. The parallels between characteristics of stem cells and aggressive cancer cells extend beyond metabolic rewiring to proliferative, migratory and self-renewing properties, and the acquisition of stem cell-like features in cancer cells is closely associated with increased invasiveness and metastasis^14^,^15^. At the functional level, the activation of epithelial-mesenchymal transition (EMT) program enables metastatic dissemination of the cancer cells including the capability for self-renewal^16^. Hence, the progression of EMT and cancer stem cell-like features in tumors confer resistance to therapeutics and are associated with poor prognosis^17^.

The parallels between highly aggressive cancers and stem cells raise the possibility that the genes regulating cellular metabolic pathways, especially fatty acid metabolism, may be altered during metastatic progression of human cancers. Here, we studied the genetic alterations in metabolic genes associated with metastatic progression using the multi-cancer Translation of the Cancer Genome Atlas (TCGA) pan-cancer datasets. We adapted a hypothesis-driven approach, wherein, we constructed a literature curated pathway model of genes altered in cancer cells that regulate glycolysis (Warburg effect) or FA metabolism, including cellular FA uptake. Next, we identified the genes exhibiting significant accumulation of mutations or copy number alterations (CNA) in metastatic tumors as compared to primary tumors. We further established a prognostic gene signature by analyzing the gene expression patterns of metabolic genes in context of EMT progression in seven different TCGA datasets. The prognostic value of the alterations and gene signature were validated by stratification of the pan-cancer samples and evaluating the influence on survival rates. Our analysis revealed that the Warburg effect genes with significant prognostic value (*TP53, STK11*) were uniformly mutated across all tumor types (primary or metastatic). However, genes involved in cellular FA uptake (*CAV1, CD36*) and de novo lipogenesis (*PPARA, PPARD, MLXIPL*) were specifically amplified at higher frequencies in metastatic tumors. In addition, the OXPHOS gene *SCO2*, a transcriptional target of p53 was also amplified in metastatic tumors. Contrary to expectations, the expression levels of *SCO2* were higher in p53 mutation background, with significant influence on prognosis. By analyzing gene expression data, we found that *CAV1, CD36, MLXIPL, CPT1C* and *CYP2E1* were the top ranked genes associated with an EMT phenotype. Stratification of samples based on copy numbers or expression profiles of the genes identified in our analysis revealed significant influence on patient survival rates.

## Results

**Figure 1:**
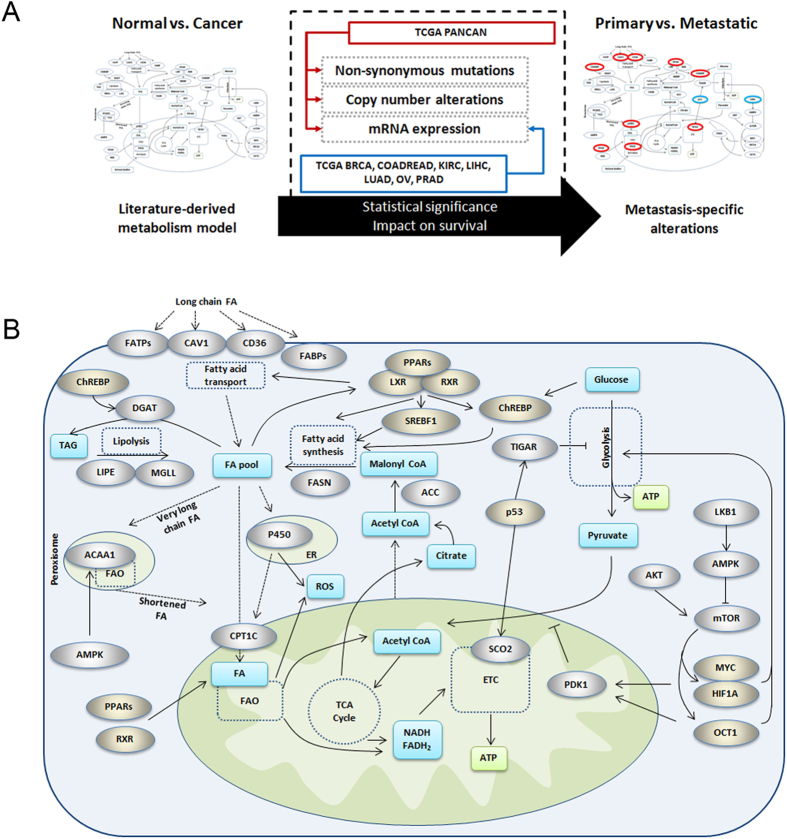
Overview of approach and metabolism alterations in cancer cells. (**A)** Schematic showing the approach and datasets used in the study to identify metabolic alterations that are relevant to metastatic progression of cancer cells. Mutation, CNA and mRNA expression data of the genes in the literature-derived model were analyzed for statistical significance in samples stratified by metastatic status. (**B)** Literature-derived metabolic pathway model showing the genetic alterations that control glucose and fatty acid metabolism in cancer cells. The genes affecting FA transport were included in the pathway as potential contributors to the FA pool. FATPs indicate FATP1-6, FABPs indicate FABP1-9, and PPARs indicate PPAR-α, -δ and -γ. Silver ovals = proteins/enzymes, gold ovals = transcription factors, blue box = metabolites, dashed lines = transport, yellow box = ATP.

**Figure 2:**
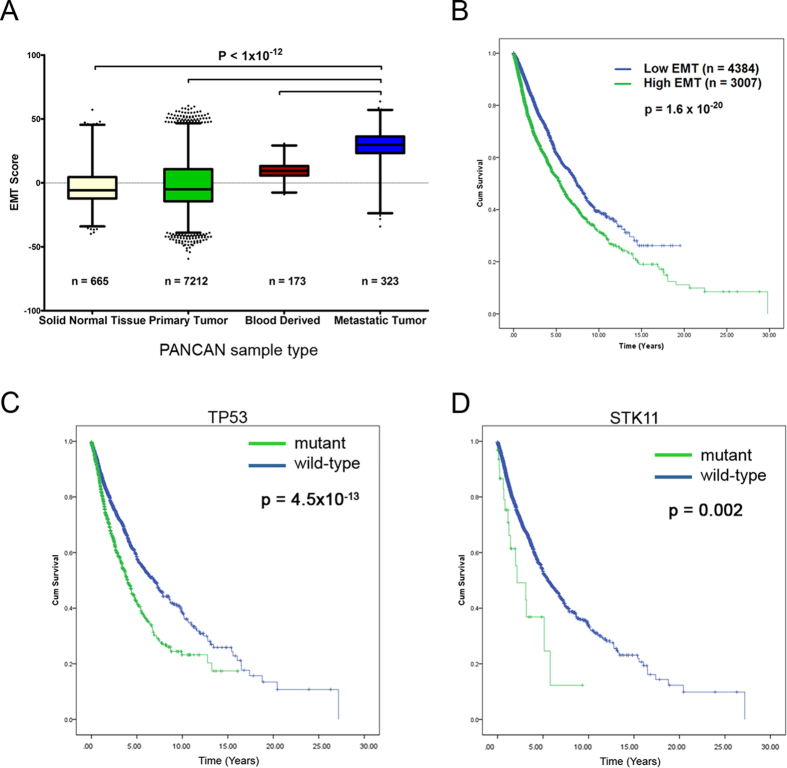
EMT scores and influence of mutations on survival. (**A**) Distribution of EMT scores in different tumor types in the pan-cancer gene expression dataset. Boxplots show mean and SEM with whiskers indicating 1–99^th^ percentile. P-values indicate significance of difference in means from Tukey’s HSD post-hoc analysis (following one-way ANOVA). (**B**) EMT score as a metric for metastatic potential and impact on patient survival rates. Kaplan-Meier survival curve for the pan-cancer patient tumors stratified according to Low EMT (< Avg. EMT score) or High EMT (≥ Avg. EMT score). Kaplan-Meier survival curve for pan-cancer tumors stratified by the mutation status of (**C**) *TP53* and (**D**) *STK11*. **(B–D).** P-value indicates significance levels from the comparison of survival curves using the Log-rank (Mantel-Cox) test.

In order to identify the metabolic genes that are significantly altered in metastatic cancer cells, we first constructed a literature-derived pathway model of genes that regulate FA metabolism and glycolysis in cancer cells (Supplementary Table 1) and analyzed for changes in mutations, copy number alterations and mRNA expression patterns that altered significantly in metastatic tumors (Fig. 1A). We further validated the clinical relevance of these alterations by their ability to predict patient survival rates. The list of candidate genes in the literature-based model (herein denoted as model genes) was restricted to genes previously reported to be significantly altered between normal and cancer cells, and included enzymes, signaling or transcriptional regulators of glucose metabolism (Warburg effect), FA oxidation, and lipogenesis (including lipolysis and esterification) (Fig. 1B)^4^,^18^,^19^,^20^,^21^. Additionally, we included the FA transport genes since elevated uptake of exogenous FAs contributes to the cellular FA pool^20^, and a recent study from our laboratory showed that elevated uptake of free fatty acids via CD36 was associated with induction of the EMT program in liver cancer cells^22^. To investigate the influence of genetic alterations in the model genes, we compared the mutation, CNA and gene expression profiles of the model genes between tumors classified as primary or metastatic in the TCGA pan-cancer datasets, or against a composite EMT-score in the TCGA datasets of individual cancers. The mRNA expression-based EMT score, consisting of relative expression levels of mesenchymal and epithelial genes, served as a proxy for metastatic status^23^. Using analysis of variance (ANOVA) test followed by Tukey’s post-hoc test, the EMT score was significantly higher in the pan-cancer metastatic tumors than the primary tumors (p = 2.1 × 10^−13^) (Fig. 2A). In addition, we evaluated the influence of the EMT score on the patient’s prognosis by classifying the samples by their mean EMT score (Fig. 2B), and found significant reduction in survival rates in the high EMT subset (p = 1.6 × 10^−20^).

### Accumulation of FA metabolism gene mutations in metastatic tumors

We evaluated the influence of non-synonymous mutations in the FA metabolism and Warburg effect genes from the pan-cancer dataset. We found that mutations in the tumor suppressors *TP53* (p = 4.5 × 10^−13^) and *STK11* (LKB1, p = 0.002) resulted in significant reduction in survival rates (Fig. 2C,D). Both *TP53* and *STK11* are among the 127 significantly mutated genes identified in the mutation landscape study of the pan-cancer cohort^24^. In addition, we evaluated the role of cumulative mutations within each model category and found the most frequently mutated genes in the Warburg effect category (Supplementary Figure 1A), however *TP53* alone contributed nearly 97% of the mutations in this group. In terms of survival rates, the presence of single or multiple mutations in the Warburg effect or FA metabolism and uptake genes significantly reduced cumulative survival (p = 2.7 × 10^−7^) (Supplementary Figure 1B). In fact, single or multiple mutations in the Warburg effect group alone significantly reduced survival rates (p = 9.7 × 10^−12^) (Supplementary Figure 1C), while mutations in the FA transport and metabolism group of genes were not significant (Supplementary Figure 1D,F). When compared to primary tumors, 50% of the FA oxidation, 41% of the lipogenesis and 24% of cellular FA uptake genes exhibited significantly higher mutation frequency in metastatic tumors, whereas none of the Warburg effect genes showed higher mutation frequency in metastatic tumors (Supplementary Table 2, Supplementary Figure 1G,H). These results highlight the fact that the Warburg effect gene mutations in *TP53* and *STK11* did not accumulate in metastatic tumors, and were associated with reduced survival rates uniformly across all tumor types.

**Figure 3:**
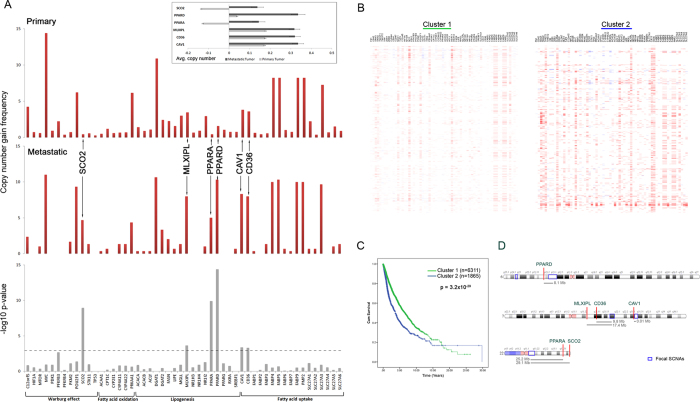
Metabolic gene CNA accumulations in metastatic tumors. (**A**) Comparison between primary and metastatic tumors for frequency of tumors with copy number gains in metabolic genes. Red bars indicate the frequency of tumors that show a copy number gain (defined as GISTIC2 normalized copy number gain > 1), grey bars indicate –log10 of the p-value from Fischer’s exact test (two-tailed) comparing copy number gain frequency of each gene between primary and metastatic tumors. Inset bar graph shows average copy numbers of the six significant genes in primary and metastatic tumors. (**B**) Heatmaps showing copy number alterations in pan-cancer tumors split into clusters. The tumors were clustered (K-means, *k* = 2) according to the copy number profiles of the 6 genes with significant accumulation of copy number gains in metastatic tumors (*SCO2, MLXIPL, PPARA, PPARD, CAV1 and CD36*). (**C**) Kaplan-Meier survival curve for pan-cancer tumors stratified by the two copy number distribution clusters. P-value indicates significance levels from the comparison of survival curves using the Log-rank (Mantel-Cox) test. **(D)** Ideograms depicting the genomic location of the six significant copy number gains. Blue boxes indicate the cytogenetic bands that were identified as focal somatic number alterations gain peaks in the GISTIC analysis of TCGA data across all cancers, and the gray bars show the distance between the six genes to the nearest gain peak.

We next evaluated the accumulation of CNAs in metastatic tumors (Supplementary Table 3). For this analysis, we used the GISTIC2 (Genomic Identification of Significant Targets in Cancer, version 2.0) calls from the pan-cancer CNA dataset to calculate the frequency of patients with either a copy number gain or copy number loss, defined as a GISTIC2 call ≥1 or ≤1, respectively. We used the Fisher’s exact test to compare if the gain or loss frequencies were significantly different between metastatic and primary tumors (Fig. 3A). We did not observe significant difference in copy number loss frequencies of any gene between primary and metastatic tumors. However, 6 genes were amplified at significantly higher frequencies in metastatic tumors: *SCO2* (p = 1.1 × 10^−9^), *MLXIPL* (p = 2.1 × 10^−4^), *PPARA* (p = 1.2 × 10^−10^), *PPARD* (p = 4 × 10^−15^), *CAV1* (p = 3.4 × 10^−4^) and *CD36* (p = 3.5 × 10^−4^). To further assess the prognostic role of these genes, we classified the pan-cancer tumors into two clusters using their CNA profiles (Fig. 3B). The samples were classified as low copy number (Cluster 1) or high copy number (Cluster 2) based on the 6-genes, identified in the previous step. A Kaplan-Meier curve analysis of the two clusters indicates a reduction in survival rates in the high copy number cluster (p = 3.2 × 10^−29^) (Fig. 3C). When compared to the known regions of focal somatic copy number alterations, none of the six genes overlapped with focal peak regions of amplification^25^ (Fig. 3D). However, *CAV1* (p = 8.3 × 10^−6^) and *CD36* (p = 0.01) were both significantly amplified across all cancers according to the genome-wide GISTIC analysis.

**Figure 4:**
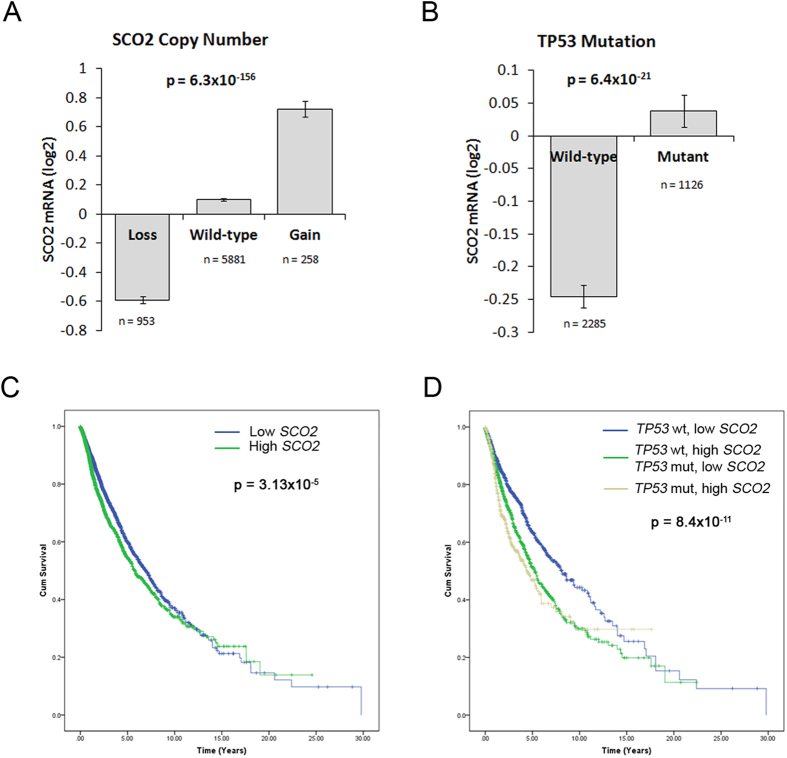
*SCO2* overexpression in *TP53* mutation background. (**A)** Bar graph showing average *SCO2* expression levels in tumors loss or gain in copy number compared to somatic levels in the pan-cancer dataset. (**B)** Bar graph showing average expression of *SCO2* in context of p53 mutation background. P-values in (**A**,**B)** indicate significance level from one-way ANOVA. **(C)** Kaplan-Meier survival curves indicating the influence of tumor stratification on the basis of low (n = 4366) or high (n = 3025) *SCO2* expression in the pan-cancer dataset. (**D)** Kaplan-Meier survival curves showing the influence of wild-type p53 and low *SCO2* expression (n = 1561), wild-type p53 with high *SCO2* expression or mutant p53 with low *SCO2* expression (n = 1269), and mutant p53 with high *SCO2* expression (n = 495) on the survival rates in the pan-cancer dataset. P-values in (**C**,**D)** indicate significance levels from the comparison of survival curves using the Log-rank (Mantel-Cox) test.

The carbohydrate response element binding protein ChREBP encoded by *MLXIPL* transcribes lipogenic enzymes *LPK*, *ACC1* and *FASN* and gluconeogenesis enabling enzyme *G6Pase* in response to increased levels of carbohydrates^26^,^27^. While the fatty acid synthesis reaction requires NADPH, the switch to aerobic glycolysis in cancer cells promotes the pentose phosphate pathway, which serves as the major source of cellular NADPH^7^. The PPAR transcription factors (*PPARA, PPARD*) transcribe genes that regulate a number of lipid metabolism pathways including lipolysis, fatty acid uptake, oxidation and lipogenesis^28^, and together with the amplification of the fatty acid transporters, *CAV1* and *CD36,* would result in elevated synthesis and accumulation of fatty acids^20^. The expression of *CAV1* has been previously discussed in the context of tumor growth^29^, and has been found to be associated with metastasis and poor prognosis of prostate^30^, pancreatic metastasis^31^ and gastric cancer^32^. While not as extensively studied, the fatty acid translocase encoding *CD36* has been recently reported to be associated with the progression of liver cancer^22^, breast cancer^33^, glioblastoma^34^ and the *in vitro* proliferation of sarcoma and breast cancer cells^35^. The p53-regulated *SCO2* gene, encodes a protein that, together with *SCO1*, regulates the assembly of electron transport chain-associated cytochrome C oxidase complex^36^. Mutations in *TP53* downregulate the transcription of *SCO2*, thereby preventing the assembly of this critical OXPHOS complex and increasing the cell’s reliance on glycolysis for ATP^37^. However, we found that the *SCO2* gene was frequently amplified in metastatic tumors and these alterations corresponded to the mRNA levels of *SCO2* (Fig. 4A). Contrary to expectations, *SCO2* mRNA levels were significantly higher in tumors bearing *TP53* mutations (Fig. 4B) and higher *SCO2* expression was associated with poorer prognosis (Fig. 4C). Additionally, the combined impact of p53 mutation and high *SCO2* expression resulted in the lowest survival rates compared to unaltered state of both genes or altered state of one of the two genes (Fig. 4D). It was reported that both mutations and overexpression of *SCO2* gene can delay the assembly of cytochrome C oxidase on the mitochondrial membrane^38^. However, it remains to be identified whether *SCO2* amplification serves as a mechanism to bypass the influence of p53 mutation on OXPHOS, or whether *SCO2* amplification further disrupts OXPHOS thereby driving the cell towards aerobic glycolysis.

In addition to the literature-curated model genes, we also analyzed the influence of the lipid and carbohydrate metabolism gene sets obtained from the REACTOME database. We found that copy number gains in only 2 out of the 471 lipid (*CYP27B1, SUMF2*) and 1 out of 235 carbohydrate (*PHKG1*) metabolism genes had a significant impact on survival rates. Further, mutations in only 2 lipid (*PIK3CA, PRKD1*) metabolism genes influenced survival rates while none of the carbohydrate metabolism gene mutations had a significant impact. Of the significant CNA and mutation genes, only *PIK3CA* mutations frequency varied significantly between primary and metastatic tumors. (Supplementary Figure 2)

### Establishing a metabolic gene expression signature associated with metastatic progression

We performed an association analysis between the mRNA expression profiles of the model genes and the EMT score for several epithelial cancers from the TCGA database: breast cancer (BRCA), colorectal adenocarcinoma (COADREAD), kidney renal clear cell cancer (KIRC), liver hepatocellular carcinoma (LIHC), lung adenocarcinoma (LUAD), ovarian cancer (OV) and prostate adenocarcinoma (PRAD). Using the nearest neighbor algorithm based on Pearson’s correlation with stringent family-wise error rate control (Bonferroni’s FWER), we identified the model genes with either significant positive or negative association with EMT score (Supplementary Tables 4–10).

**Figure 5:**
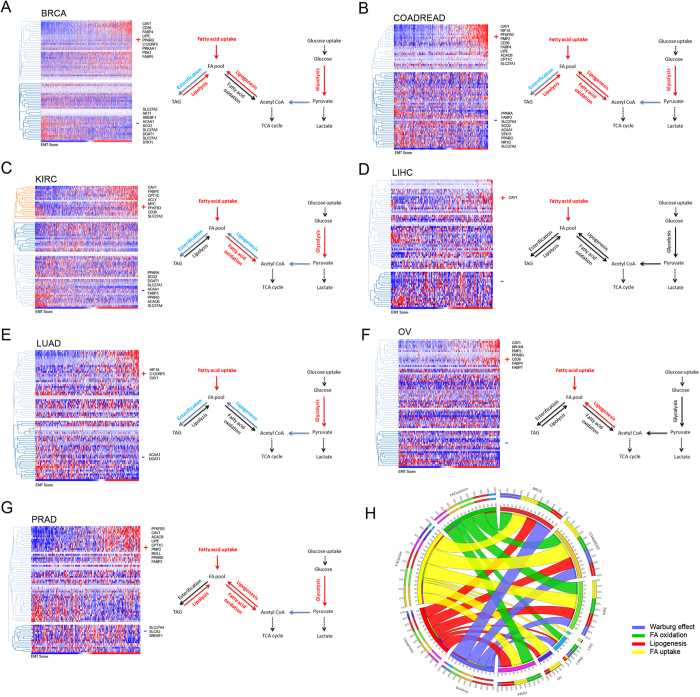
Association between metabolic gene expression and metastatic potential in multiple cancers. Heatmaps showing expression profiles of EMT and metabolic genes in **(A)** Breast invasive carcinoma, BRCA (n = 1037) (**B)** Colorectal adenocarcinoma, COADREAD (n = 352) **(C)** KIRC (n = 518) (**D)** Liver hepatocellular carcinoma LIHC (n = 191) (**E)** LUAD (n = 230) **(F)** Ovarian serous cystadenocarcinoma, OV (n = 261) **(G)** Prostate adenocarcinoma, PRAD (n = 236). The tumor samples (X-axis) are ordered according to EMT score shown in bottom row of each heatmap and genes (Y-axis) are arranged in hierarchical clusters (average linkage method, Pearson’s correlation distance metric). Genes with significant positive (+) or negative (−) correlation with EMT score are listed next to the heatmap, along with a schematic of the metabolic processes predicted to be altered on the basis of significant correlation in each cancer type. (**H)** Circos diagram showing the relationship between the percentage of significant EMT-associated genes within each cancer type and the percent contribution to the total number of significant EMT-associated genes within each model category.

An intriguing generalized pattern emerged from this analysis. We found that elevated expression of fatty acid uptake genes, *CAV1* and *CD36,* were consistently associated with a high EMT score across all cancers. We also observed cancer type-specific alterations in the pathways. For instance, in ovarian cancer, the Warburg effect genes typically altered in all other cancer types were not associated with the EMT score. Similarly, the expression levels of FA oxidation and lipogenesis genes were not associated with the EMT score in lung cancer. (Supplementary Figure 3). As the activation of aerobic glycolysis is evident in neoplastic tissues independent of the extent of vascularization^39^, one possible explanation may be that the alterations related to Warburg effect are early events in pathogenesis of ovarian cancer^40^, whereas the activation of lipogenesis has been previously reported in neoplastic lung tumors^41^. These patterns are reflected in the simplified schematics in Fig. 5A–G, showing the expected influence of the genes significantly associated with EMT score in each cancer type. Here, we note that the processes that could elevate the FA pool were strongly associated with the EMT score. For instance, we observe significant negative correlation between the EMT score and the esterification gene (*DGAT1*) in 4 cancer types (BRCA, COADREAD, KIRC, LUAD), and significant positive correlation with lipase (*LIPE*) in 3 cancer types (BRCA, COADREAD, PRAD) (Supplementary tables 4–10). Furthermore, the appearance of *CAV1* and *CD36* across all the cancer types in this study clearly supports the role of elevated cellular FA uptake and accumulation in the progression and maintenance of metastatic tumors. Overall, we observed that the genes in fatty acid uptake pathway constituted the largest percentage of significant genes associated with EMT score among the four categories analyzed (Fig. 5H). Additionally, the largest fractions of significant Warburg effect genes were contributed by the breast cancer dataset, whereas the colorectal cancer contributed the largest fraction of significant cellular FA uptake genes. The breast and colorectal cancer datasets together contributed nearly half of all the significant genes in this analysis.

**Figure 6:**
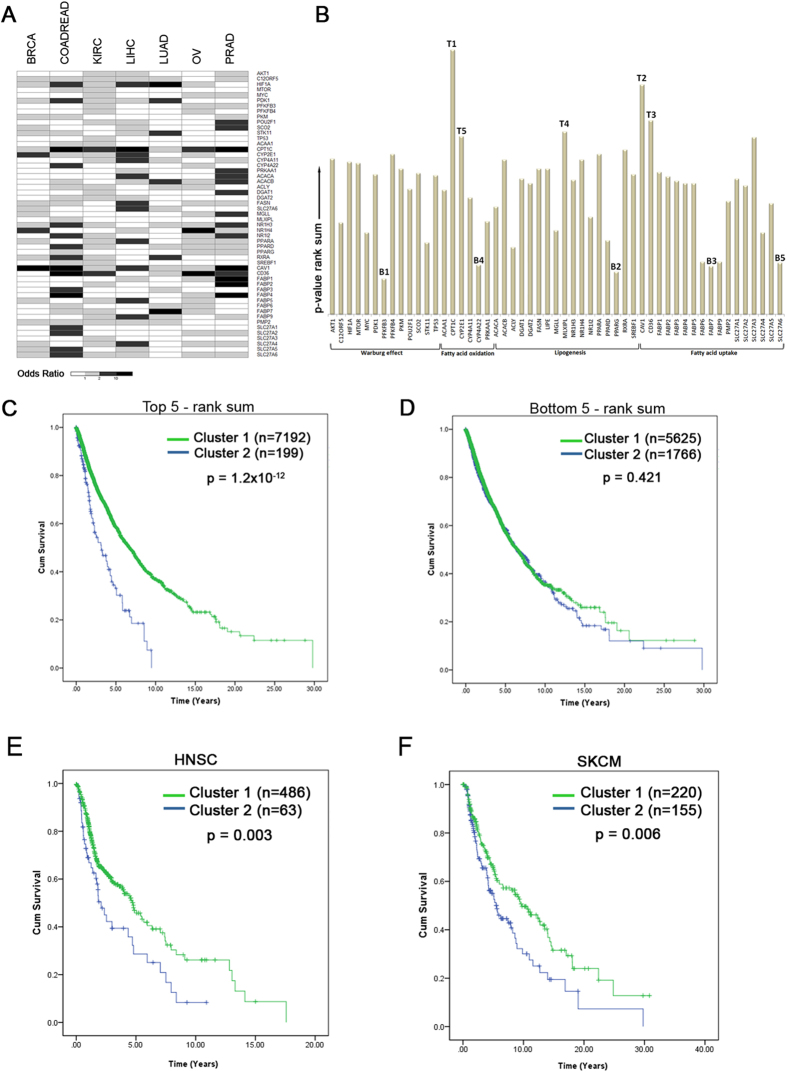
Influence of fatty acid metabolism gene signature on patient survival. (**A)** Odds ratio obtained from logistic regression analysis demonstrating association between individual metabolism genes and EMT (low or high) across the seven cancer types, with shades of grey indicating increasing odds ratio. (**B**) Bar graphs showing cumulative rank sum of odds-ratio –log10 p-value of individual genes across all seven cancer types. T1-5 and B1-5 indicate the five top and bottom ranked genes respectively (**C,D).** Kaplan-Meier survival curve for pan-cancer tumors stratified by two mRNA expression clusters. The sample clusters were obtained by K-means (*k* = 2) using (**C**) top 5 genes (*CPT1C, CAV1, CD36, MLXIPL, CYP2E1*) or (**D**) bottom 5 genes (*PFKFB3, PPARG, FABP7, CYP4A22, SLC27A6*) from the rank-sum analysis. P-value indicates significance levels from the comparison of survival curves using the Log-rank (Mantel-Cox) test. **(E,F)** Kaplan-Meier survival curves for (**E**) head & neck cancer (HNSC) or (**F**) melanoma (SKCM) tumors stratified by the mRNA expression profiles of the top-ranked genes identified in the previous analysis.

We next derived a gene signature using a logistic regression based approach to calculate the odds ratio for the influence of each gene on EMT and patient prognosis in individual cancer types (Fig. 6A, Supplementary Table 11). The genes were ranked based on their influence on EMT score across all the cancer types using a cumulative rank-sum metric obtained from the odds ratio p-values (Fig. 6B). Based on the cumulative ranks, the top 5 genes identified were *CPT1C, CAV1, CD36, MLXIPL,* and *CYP2E1.* The fatty acid uptake genes *CAV1* and *CD36,* as well as the carbohydrate response element binding protein encoding *MLXIPL*, also exhibited significant accumulation of copy number gain in the pan-cancer metastatic tumors (Fig. 3A). *CPT1C* was found to mediate the uptake of long-chain fatty acid across the mitochondrial membrane to activate β-oxidation, and promote tumor adaptation under metabolic stress conditions^42^. *CYP2E1* encodes the microsomal P450 2E1 protein that is involved in xenobiotic, acetone and fatty acid metabolism. *CYP2E1* binds to saturated fatty acids (C_10_-C_20_), arachidonic acid and linoleic acids, and catalyzes hydroxylation of fatty acids in the microsomal ω-oxidation pathway^43^. The expression levels of *CYP2E1* were found to be correlated with accumulation of fats in animal models as well as in human non-alcoholic fatty liver disease patients, and resulted in elevated cellular reactive oxygen species (ROS) levels as a byproduct of *CYP2E1*-mediated fatty acid metabolism^44^,^45^. Additionally, *CYP2E1* alterations were found to be correlated with the development of malignant tumors^46^,^47^. Using the 5-gene signature to classify the pan-cancer samples based on mRNA levels found the high expression cluster (Cluster 2) resulted in dramatic decrease in the survival rates of the patients (p = 1.2 × 10^−12^) (Fig. 6C). In contrast, the clusters based on the bottom ranked genes did not influence the survival rates (p = 0.421) (Fig. 6D). For validation, the prognostic value of the 5-gene signature were used to clustered two independent TCGA datasets that were not used to generate the gene signature – head and neck squamous cell carcinoma (HNSC) and skin cutaneous melanoma (SKCM). The clusters with high expression of the signature genes resulted in significant decrease in survival rates of both head & neck cancer (p = 0.003) and melanoma (p = 0.006) patients. Thus, the metabolic gene signature identified in the analysis successfully predicted poor prognosis in unrelated cancer dataset, further supporting the role of fatty acid uptake and metabolism in metastatic progression of cancers.

In this study, the cellular FA uptake genes *CAV1* and *CD36* were found to be amplified in metastatic tumors, their gene expression patterns were consistently associated with EMT scores across multiple cancer types and emerged as members of the predictive gene signature. Given the previously undocumented role of cellular FA uptake in cancer progression and metastasis, we stratified the pan-cancer samples based on the gene expression profiles of *CAV1* and *CD36.* We then performed gene-set enrichment analysis with oncogenic gene signatures on the high and low expression clusters. Intriguingly, only 1 out of 187 oncogenic gene signatures were enriched in the low expression cluster, whereas nearly 29% (54 out of 187) of all oncogenic signatures were enriched in the high expression cluster (Supplementary Table 12), thereby confirming the strong association between elevated expression of cellular FA uptake genes with cancer progression.

## Discussion

In recent years, deregulated glycolysis and fatty acid metabolism has been linked with resistance to chemotherapeutics in multiple cancer types^48^. Consequently, the regulators of cancer cell metabolism have emerged as attractive therapeutic targets and primarily include genes that are significantly altered between normal and cancer cells, such as glucose transporters (*GLUT1*, *GLUT4*), hexokinase (*HK1*), pyruvate kinase (*PKM2*) and fatty acid synthase (*FASN*)^49^,^50^. Given the prognostic impact of the activation of EMT program and metastasis, the objective of this study was to identify the metabolic alterations that are not only relevant to carcinogenesis, but are acquired during metastatic progression of cancer cells. Supplementary Figure 4 provides a summary of the genes with prognostic consequences that are significantly altered between primary and metastatic tumors (highlighted in red).

One of the prime requirements of rapidly replicating malignant cells is fulfilling increased energy demands. We found that mutations in the tumor suppressor genes that control Warburg effect, *TP53* and *STK11,* uniformly influenced survival rates across all tumors. From a therapeutics perspective, gene therapy with *TP53* (rAd-p53, Ad5CMV-p53), and antidiabetic medication metformin, which indirectly activates *STK11*/AMPK activity^51^, are currently being evaluated under multiple clinical trials for various cancer types (source: ClinicalTrials.gov). However, the mutation frequencies were not elevated in metastatic tumors, suggesting that the switch to glycolysis may occur during the early stages of cancer progression. Glycolysis produces ATP at greatly reduced efficiency as compared to mitochondrial OXPHOS and in vitro studies with malignant cancer cells have reported that OXPHOS may still contribute to the bulk of ATP produced by cancer cells depending on the availability of oxygen^52^,^53^. In such a scenario, alternate pathways must provide the acetyl CoA that is metabolized in the TCA cycle to produce NADH and FADH_2_ for ATP synthesis through OXPHOS. β-oxidation of fatty acids could be advantageous to cancer cells by providing a supply of acetyl CoA, NADH and FADH_2_ during aerobic glycolysis but would require a steady supply of the substrate, free fatty acids. The intracellular pool of free fatty acids can be positively influenced by lipolysis of stored triglycerides, elevated uptake of fatty acids from the environment, or lipogenesis using carbon from carbohydrates, amino acids or oxidized fatty acids. While the upregulation of *FASN* is well documented in cancer cells, we did not observe a significant change in metastatic cells. Instead, we observed significant amplification of *PPARA* and *MXIPL* in metastatic tumors which could upregulate the transcription of lipogenesis genes. Moreover, we found amplifications in the cellular FA uptake genes *CAV1* and *CD36* (MLXIPL, CAV1 and CD36 also impact patient survival rate individually, see Supplementary Figure 5). While recent studies showed that cancer cells activate lipid scavenging pathways during nutrient starvation^54^,^55^, our results suggest that metastatic cancer cells might increase the cellular FA pool by elevating the uptake of exogenous FAs. Furthermore, we observed gain of *CPT1C* and *CYP2E1* expression which may be indicative of increased mitochondrial β-oxidation and microsomal ω-oxidation, respectively, in metastatic tumors.

In addition to providing energy, the elevated oxidation of FAs also affects cellular redox status by elevating the levels of reactive oxygen species (ROS) and by reducing the NAD^+^/NADH ratio^56^. ROS are well known as potent DNA-damage inducing agents that not only increase genomic instability to drive malignant transformation, but also influence signaling pathways that increase proliferation and confer resistance to apoptosis^57^,^58^,^59^. Elevated NADH levels induce proliferation and migration by repressing transcription of E-cadherin by regulating the activity of CtBP co-repressor^60^,^61^. Recently, reduced NAD^+^/NADH ratios were shown to drive the metastatic progression of MDA-MB-435 and MDA-MB-231 breast cancer xenografts by activating AKT/mTOR and autophagy signaling pathways while increasing the NAD^+^/NADH ratio inhibited tumor growth^62^.

We have identified a group of FA metabolism and uptake genes that are specifically altered in metastatic tumors, and demonstrated the clinical relevance of these alterations based upon their influence on patient survival rates. In addition, we suggest that the alterations driving the switch to aerobic glycolysis or the Warburg effect occur uniformly across primary and metastatic tumors. Further functional studies are warranted to understand the precise role of these alterations in tumor progression and their association with the activation of EMT program. Given the poor post-therapy survival rates in patients with advanced, metastatic tumors, the genes and associated pathways identified in this study could serve both as biomarkers as well as new therapeutic targets in future studies.

## Methods

### Cancer-specific metabolic pathway model

We selected the enzymes and signaling or transcriptional regulators of glucose or fatty acid metabolism based on the criteria that they are altered in cancer cells. For glucose metabolism, we included the genes that are involved in the switch to aerobic glycolysis or Warburg effect in cancer cells^4^,^18^. This process is controlled by the oncogenes hypoxia inducible factor 1 alpha or HIF1α (*HIF1A*), avian myelocytomatosis viral oncogene homolog or c-MYC (*MYC*) and POU domain, class 2, transcription factor 1 or OCT1 (*POU2F1*) that control the transcription of glycolytic enzymes, in addition to *PDK1* (pyruvate dehydrogenase kinase 1) that prevents entry of pyruvate into TCA cycle by inhibiting pyruvate dehydrogenase. The glycolytic enzymes activated by HIF1α include *PFKFB4* (6-phosphofructo-2-kinase/fructose-2,6-bisphosphatase 4), which diverts glucose towards the pentose phosphate pathway, and *PFKFB3*, which promotes glycolysis. RAC-alpha serine/threonine-protein kinase 1 or AKT1 activates mechanistic target of rapamycin or mTOR, which in turn activates HIF1A, whereas the tumor suppressor liver kinase B1 (*STK11*) activates AMP-activated protein kinase or AMPK (*PRKAA1*) which can inhibit the activity of mTOR. Activation of mTOR signaling results in HIF1α activation and expression of c-MYC and OCT1, thereby, enabling the glycolytic switch. *PKM* (Pyruvate kinase) catalyzes the final step of glycolysis converting phosphoenol pyruvate to pyruvate generating one molecule of ATP. However, the M2 isoform of pyruvate kinase expressed at high levels in cancer cells inhibits the catalytic conversion step, and promotes the utilization of the substrates through the pentose phosphate pathway. The tumor suppressor p53 controls glycolysis by regulating the transcription of TP53-induced glycolysis and apoptosis regulator or TIGAR (*C12ORF5*) and *SCO2* which regulates the assembly of ETC associated cytochrome C oxidase complex.

For fatty acid metabolism, in addition to the de novo lipogenesis and fatty acid oxidation genes altered in cancer cells, we included genes that may alter the availability of fatty acids to the cancer cells from external sources^19^,^20^,^21^. ATP-citrate lyase (*ACLY*) controls the synthesis of acetyl-CoA from citrate, thus, linking carbohydrate metabolism to fatty acid synthesis. The conversion of acetyl-CoA to malonly-CoA is catalyzed by acetyl-CoA carboxylase complex (*ACACA, ACACB*). Fatty acid synthase (*FASN*) then catalyzes the condensation of malonyl-CoA and acetyl-CoA into fatty acid. The master regulator of fatty acid synthesis genes, Sterol regulatory element-binding proteins or SREBP (*SREBF1*), is in turn transcriptionally regulated by the Liver X Receptor (*NR1H3*) and peroxisome proliferator-activated receptor alpha or PPAR-α (*PPARA*) that function as a heterodimer with retinoid X receptor (*RXRA*). Carbohydrate-responsive element-binding protein or ChREBP (*MLXIPL*) regulates the transcription of lipogenic enzymes *FASN, ACACA* and *ACACB* and is activated by elevated glucose levels. The rate limiting step in mitochondrial oxidation is the transport of fatty acids across the membrane through carnitine palmitoyltransferase 1 (CPT1). Among the different isoforms, the brain isoform *CPT1C* is expressed at elevated levels in cancer cells to increase FA oxidation and ATP production. PPAR-α induces expression of Cytochrome P450 2E1 or *CYP2E1* which is involved in the ω-oxidation of fatty acids as a part of the microsomal cytochrome P450 complex. In peroxisomes, acetyl-CoA C-acyltransferase (*ACAA1*) catalyzes the final step of β-oxidation yielding acetyl-CoA whereas the cytochrome P450 4A enzymes (*CYP4A11, CYP4A22*) catalyze microsomal ω-hydroxylation of fatty acids in the endoplasmic reticulum. The dicarboxylic acid generated as a result of ω-oxidation of fatty acids can serve as substrates for β-oxidation or serve as PPAR ligands. The balance between stored lipids and free fatty acids are regulated by diglyceride acyltransferase enzymes (*DGAT1, DGAT2*) which catalyze the esterification of fatty acids to triglycerides, whereas hormone-sensitive lipase (*LIPE)* and monoacylglycerol lipase (*MGLL)* produce free fatty acids by hydrolyzing triglycerides and mono-acylglycerids, respectively. Peroxisome proliferator-activated receptor gamma or PPAR-γ (*PPARG*) promotes lipid storage by regulating expression of esterification enzymes and opposes lipid synthesis, whereas peroxisome proliferator-activated receptor delta or PPAR-δ (*PPARD*) promotes fatty acid transport and oxidation. The poly unsaturated fatty acid activated farnesoid X receptor or FXR (*NR1H4*) inhibits the activity of LXR and regulates *SREBF1*, *PPARA* and *PPARG*, and inhibits lipogenesis. Pregnane X receptor or PXR (*NR1I2*) induces the transcription of fatty acid transport protein CD36 but suppress expression of FA oxidation genes *PPARA* and *ACAA1*. The accumulation of fatty acids in the cells from exogenous sources is mediated by the transport proteins caveolin 1 (*CAV1*), cluster of differentiation 36 (also known as FAT, fatty acid translocase) *CD36* and fatty acid transport proteins FATPs (*SLC27A1-6*). In addition, the intracellular trafficking of fatty acids and their interactions with nuclear receptors like PPARs and LXR are regulated by fatty acid binding proteins FABPs (*FABP1-7, PMP2, FABP9*).

### Mutation analysis

Pan-cancer gene-level somatic mutations data was obtained from the Cancer Browser (PANCAN AWG version 2014-08-22). The pan-cancer samples were divided into two groups: primary and metastatic based on sample type information.

We used the Fisher’s exact test to identify the mutations with significantly different frequency in metastatic samples compared to primary tumor samples. For each gene, the test was applied to a 2 × 2 contingency table of expected and observed mutation frequency for metastatic and primary tumor samples. The observed mutation frequency of a gene in each group were calculated as (number of samples with non-synonymous mutations in the group)/(total number of samples in group). Expected mutation frequencies for each gene were calculated as the product of mutation frequency of the gene in the complete dataset (metastatic + primary tumor) and total number of samples in a group. Genes were considered significant with a p-value cut-off set at 0.05 with stringent false discovery rate control (Bonferroni’s method). Basal mutation frequencies were calculated as the (cumulative mutation frequency of all genes in the PANCAN dataset)/(total number of genes x total number of samples).

To evaluate the influence the mutations on survival, we generated Kaplan-Meier survival curves by stratifying the patients in the complete pan-cancer dataset into two groups: no mutation and mutation. The significance of differences in survival rates were calculated using the log-rank (Mantel-Cox) test, with p-value <0.05 considered significant. The combined effects of multiple mutations in a sample were assessed by first obtaining the sum of mutations in all genes within a category. The samples were then stratified into three groups: no mutations, one mutation or multiple mutations, and the significance of differences in survival rates were calculated using the log-rank test, with p-value <0.05 considered significant.

### Copy number variation analysis

Pan-cancer gene-level somatic copy number alterations data (Genomic Identification of Significant Targets in Cancer, version 2.0 or GISTIC2 normalized) were obtained from Cancer Browser (PANCAN version 2014-08-22). We discretized the copy number data of each gene in a sample by binning the copy number calls as gain or loss using a threshold of ≥1 to denote samples with copy number gain and ≤−1 to denote samples with copy number loss.

The copy number gain or loss frequencies for each by tumor type were calculated as (number of samples with copy number gain or loss in a group)/(total number of samples in a group). Expected CNA frequencies were calculated as the product of dataset CNA frequency and total number samples in a group. For each gene, a 2 × 2 contingency table of expected and observed CNA frequencies for metastatic and primary tumor samples were analyzed with Fisher’s exact test, with a p-value cut-off set at 0.05 with stringent false discovery rate control (Bonferroni’s method).

To assess the combined influence of the CNAs with significant accumulation in metastatic tumor samples, we stratified the samples in the complete pan-cancer dataset by grouping the samples in to two homogenous subsets based on the CNA profiles of the six significant genes using the K-means clustering algorithm (Euclidean distance metric). The resulting clusters with high copy numbers and low copy numbers of the six genes were then used to generate survival curves using the Kaplan-Meier method and the significance of differences in survival rates were determined using log-rank (Mantel-Cox) test with p-value cut-off set at 0.05. The influence of individual genes were determined by stratifying the samples as copy number gain/loss or no change, and significance of difference in survival rates were obtained with log-rank test with p-value cut-off set at 0.05.

We used the tumorscape database to assess if the six significant genes obtained in our analysis overlapped with the known peaks of somatic CNA across all cancers^25^. The ideograms depicting the loci with known somatic CNA peaks and genomic location of the six significant genes were created using the NCBI genome decoration tool.

### Gene expression analysis

The TCGA pan-cancer RNAseq dataset (Illumina HiSeqV2, version 2014-08-28, RSEM normalized) and all clinical information files were retrieved using the Cancer Browser^63^,^64^. For individual TCGA cancer types, RNAseq datasets (Illumina HiSeqV2, version 2014-08-28, RSEM normalized) were retrieved from cBioPortal for cancer genomics^65^. Next, we log2 transformed all mRNA expression data and merged survival data from the clinical information file matched by sample ID.

We classified the genes based on the metastatic potential of each sample denoted by the EMT score. We first calculated the EMT score for each tumor sample in the pan-cancer or individual cancer datasets using the formula: EMT score = Sum of mesenchymal gene expression (*CDH2, FN1, SNAI1, SNAI2, VIM, TWIST1, TWIST2, ZEB2, ZEB2*) - Sum of epithelial gene expression (*CDH1, CLDN4, CLDN7, MUC1, TJP3*). To demonstrate the utility of EMT score as indicator of metastatic potential, we compared the average EMT scores across different pan-cancer subtypes (normal, primary, blood derived and metastatic) using the Tukey’s HSD post-hoc test following one-way ANOVA. We further stratified the pan-cancer samples as “low EMT” if EMT score < Avg. EMT score or “high EMT” if EMT score ≥ Avg. EMT score, and assessed the effect on survival rates using the log-rank test. To identify the genes that were associated with activation of the EMT program in the individual cancers, we classified the genes based on their similarity to the EMT score using the nearest neighbor algorithm. The distances were calculated using Pearson’s correlation coefficient (family wise error rate controlled, Bonferroni’s method), and the significant positive and negative neighbors were used to assess the overall effect on metabolism in samples in context of EMT.

We further evaluated if the expression levels of the individual genes can predict the stratification of the samples in a given cancer type by EMT score. We classified the samples as low or high EMT based on the average EMT score. Using this binary classification of the samples as the dependent variable, we analyzed the ability of all model genes to predict the classification using logistic regression analysis. For the logistic regression analysis, we used the likelihood ratio method to obtain the variables with significant association with EMT score indicated by the odds ratio (exponentiation of beta coefficient) and 2-tailed p-values from Wald chi-square test of significance. The −log10 transformed p-values were then used to assign ranks in each cancer type (lowest rank = highest p-value). We then summed ranks across all cancer types to obtain a cumulative rank-sum for each gene. Next, we retrieved the top and bottom 5 genes with the lowest and highest cumulative rank-sum, respectively, and clustered the samples (K-means, k = 2) based on the expression profiles of the two gene signatures. The sample stratification based on the gene signatures were used to assessed the survival rates of the patients using Kaplan-Meier curve analysis with the log-rank (Mantel-Cox) test.

### Gene set enrichment analysis

Gene set enrichment analysis^66^ was performed using the GenePattern platform for bioinformatics^67^, using oncogenic gene signatures. Phenotypic classes were defined by clustering pan-cancer samples (K-means, k = 2) based on gene expression profiles of *CAV1* and *CD36,* and the enrichments were evaluated in the complete pan-cancer gene expression dataset. Gene sets with enrichment p-values <0.05 and false discovery rates <25% were considered as statistically significant.

### Statistical analysis

Hierarchical clustering, nearest neighbor analysis and heatmap visualizations were performed using GENE-E matrix analysis platform. All other classification and statistical analysis procedures were performed using SPSS Statistics v20.

### Web Resources

cBioPortal for cancer genomics, http://www.cbioportal.org/ ; Cancer genomics browser: https://genome-cancer.ucsc.edu/ ; Tumorscape database, http://www.broadinstitute.org/tumorscape ; NCBI genome decoration tool, http://www.ncbi.nlm.nih.gov/genome/tools/gdp ; GENE-E matrix analysis platform, http://www.broadinstitute.org/cancer/software/GENE-E/ ; GenePattern bioinformatics platform, http://www.broadinstitute.org/cancer/software/genepattern ; ClinicalTrials.gov, https://clinicaltrials.gov/

## Additional Information

**How to cite this article**: Nath, A. and Chan, C. Genetic alterations in fatty acid transport and metabolism genes are associated with metastatic progression and poor prognosis of human cancers. *Sci. Rep.* **6**, 18669; doi: 10.1038/srep18669 (2016).

## Supplementary Material

Supplementary Information
