# Supplementary material for: Genetic alterations in fatty acid transport and metabolism genes are associated with metastatic progression and poor prognosis of human cancers
Source: Sci Rep. 2016 Jan 4;6:18669. doi: 10.1038/srep18669 (PMC4698658; doi:10.1038/srep18669)
Supplement: Supplementary Information [file srep18669-s1.pdf]

Genetic alterations in fatty acid transport and metabolism genes are associated with metastatic progression and poor prognosis of human cancers

Aritro Nath [1] and *Christina Chan, [1,2]

1 Genetics Program, Michigan State University, East Lansing, Michigan 48824, USA

2 Department of Chemical Engineering and Materials Science, Michigan State University, East Lansing, Michigan 48824, USA

* Corresponding author: krischan@egr.msu.edu

Suppl. Fig. 1

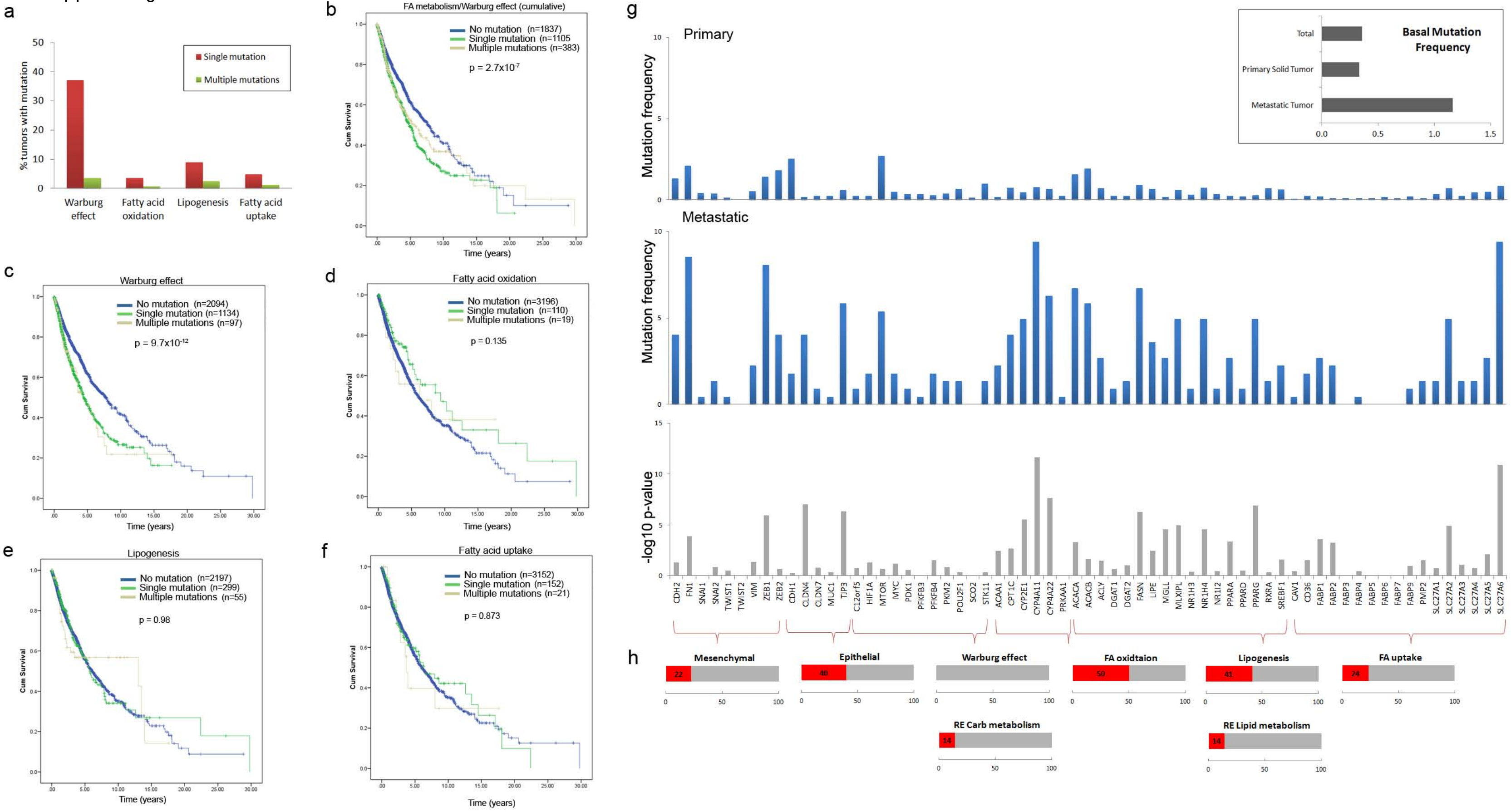
a
% tumors with mutation
Single mutation
Multiple mutations
Warburg effect
Fatty acid oxidation
Lipogenesis
Fatty acid uptake
b
FA metabolism/Warburg effect (cumulative)
No mutation (n=1837)
Single mutation (n=1105)
Multiple mutations (n=383)
p = 2.7x10-7
Cum Survival
Time (years)
c
Warburg effect
No mutation (n=2094)
Single mutation (n=1134)
Multiple mutations (n=97)
p = 9.7x10-12
Cum Survival
Time (years)
d
Fatty acid oxidation
No mutation (n=3196)
Single mutation (n=110)
Multiple mutations (n=19)
p = 0.135
Cum Survival
Time (years)
e
Lipogenesis
No mutation (n=2197)
Single mutation (n=299)
Multiple mutations (n=55)
p = 0.98
Cum Survival
Time (years)
f
Fatty acid uptake
No mutation (n=3152)
Single mutation (n=152)
Multiple mutations (n=21)
p = 0.873
Cum Survival
Time (years)
g
Primary
Mutation frequency
Metastatic
Mutation frequency
-log10 p-value
Total
Primary Solid Tumor
Metastatic Tumor
Basal Mutation Frequency
CDH2 FN1 SNAI1 SNAI2 TWIST1 TWIST2 VIM ZEB1 ZEB2 CDH1 CLDN4 CLDN7 MUC1 TJP3 C12orf5 HIF1A MTOR MYC PDK1 PFKFB3 PFKFB4 PKM2 POU2F1 SCO2 STK11 ACAA1 CPT1C CYP2E1 CYP4A11 CYP4A22 PRKAA1 ACACA ACACB ACLY DGAT1 DGAT2 FASN LIPE MGLL MLXIPL NR1H3 NR1H4 NR1I2 PPARA PPARD PPARG RXRA SREBF1 CAV1 CD36 FABP1 FABP2 FABP3 FABP4 FABP5 FABP6 FABP7 FABP9 PMP2 SLC27A1 SLC27A2 SLC27A3 SLC27A4 SLC27A5 SLC27A6
h
Mesenchymal
22
Epithelial
40
Warburg effect
FA oxidtaion
50
Lipogenesis
41
FA uptake
24
RE Carb metabolism
14
RE Lipid metabolism
14

Supplementary Figure 1: Metabolic gene mutation accumulation in metastatic tumors

A. Bar graphs showing frequency of tumors bearing single or multiple mutations in genes belonging to different metabolic categories in the pan-cancer dataset. B-F. Kaplan-Meir survival curves for pan-cancer tumors stratified according to cumulative frequencies of single or multiple mutations or no mutations in the metabolic gene category. P-values indicate significance levels from the comparison of survival curves using the log-rank (Mantel-Cox) test). G. Comparison between primary and metastatic tumors for frequency of tumors with mutations in individual metabolic genes. Red bars indicate the frequency of tumors with a non-synonymous mutation, grey bars indicate –log10 of the p-value from Fischer's exact test (two-tailed) comparing mutation frequency of a gene between primary and metastatic tumors. Inset bar graph shows basal mutation frequency of all genes in the total pan-cancer dataset and primary or metastatic tumors. H. Bars indicating the percentage of genes within each metabolic gene category with significantly altered mutation frequency between primary and metastatic tumor. Red bars indicate percentage of significant mutations, and grey bars indicate non-significant genes. The percentage of significant genes in the REACTOME carbohydrate metabolism and lipid metabolism gene sets are shown for comparison.

Suppl. Fig. 2

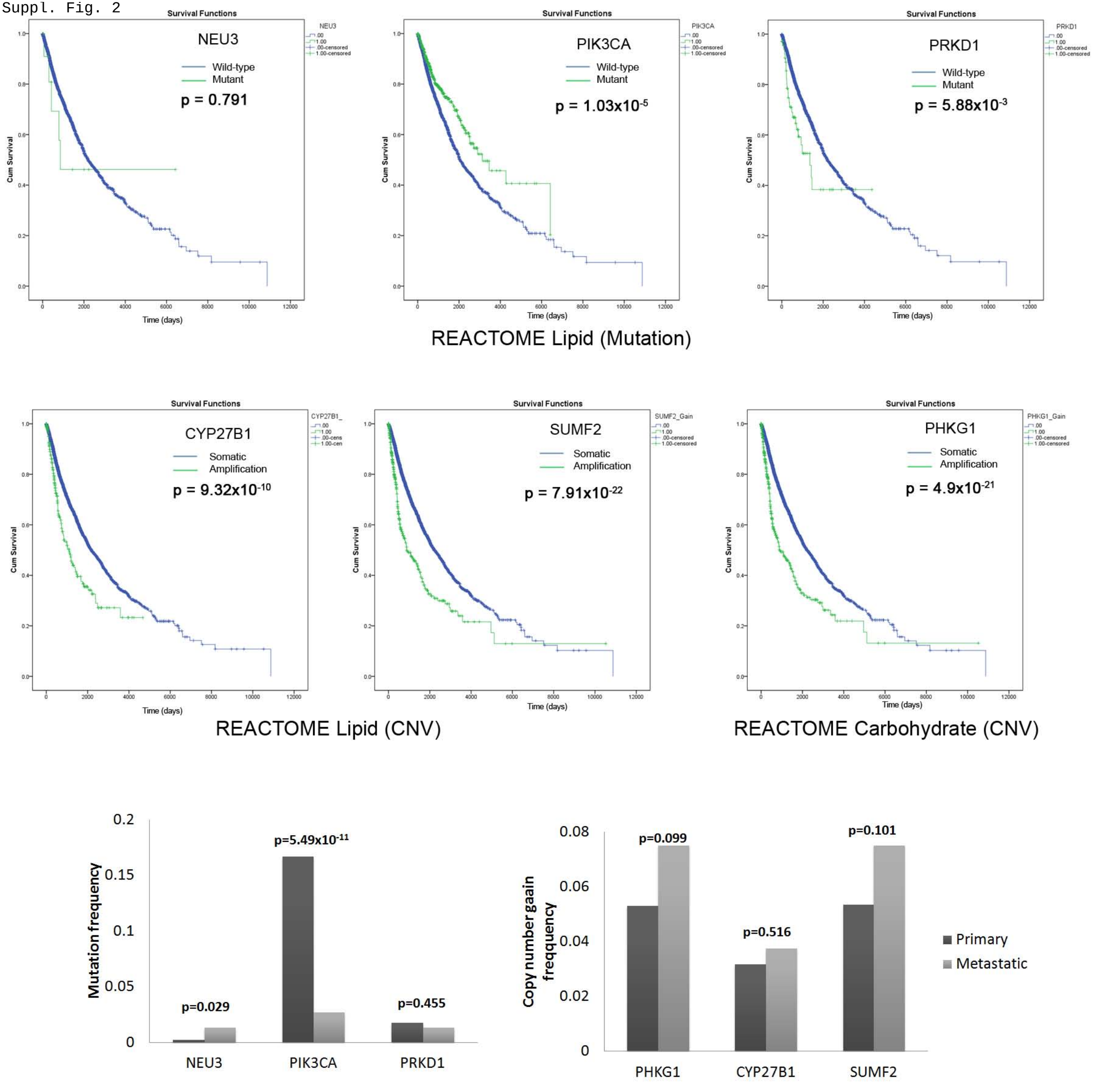
Survival Functions
NEU3
Wild-type
Mutant
p = 0.791
PIK3CA
p = 1.03x10-5
PRKD1
p = 5.88x10-3
Cum Survival
Time (days)
REACTOME Lipid (Mutation)
CYP27B1
Somatic
Amplification
p = 9.32x10-10
SUMF2
p = 7.91x10-22
PHKG1
p = 4.9x10-21
REACTOME Lipid (CNV)
REACTOME Carbohydrate (CNV)
Mutation frequency
p=0.029
p=5.49x10-11
p=0.455
NEU3
PIK3CA
PRKD1
Copy number gaain freqquency
p=0.099
p=0.516
p=0.101
PHKG1
CYP27B1
SUMF2
Primary
Metastatic

Supplementary Figure 2: Analysis of REACTOME carbohydrate and lipid metabolism gene sets

A-D Kaplan-Meir survival curves for genes with significant impact on survival rates in pan-cancer tumors stratified according to mutation frequencies or copy number gain frequencies of REACTOME carbohydrate and lipid metabolism gene sets. A. Survival curves of significant lipid metabolism mutations (*PRKD1, PIK3CA*); B. Survival curves of significant lipid metabolism amplifications (*CYP27B1, SUMF2*); C. Survival curves of significant carbohydrate metabolism amplifications (*PHKG1*) P-values indicate significance levels from the comparison of survival curves using the log-rank (Mantel-Cox) test). D-E. Bar graphs comparing mutation frequency (D) or copy number gain frequency (E) between primary and metastatic tumors of the genes with significant impact on survival rates. P-values indicate significance levels from Fisher's exact test.

Suppl. Fig. 3

		BRCA	COADREAD	KIRC	LIHC	LUAD	OV	PRAD
Warburg	AKT1	-0.19	0.04	-0.07	0.03	-0.13	-0.08	-0.08
	C12ORF5	0.27	-0.06	0.13	0.23	0.30	0.03	0.13
	HIF1A	0.24	0.44	-0.01	0.20	0.46	0.04	0.10
	MTOR	0.07	0.17	-0.02	0.05	0.10	-0.10	0.02
	MYC	0.12	-0.05	0.25	0.13	0.14	-0.10	0.01
	PDK1	0.14	-0.10	0.16	0.12	0.18	0.04	-0.03
	PFKFB3	0.04	0.35	0.21	-0.11	0.06	0.03	0.40
	PFKFB4	-0.11	-0.11	0.10	0.05	0.15	0.08	-0.20
	PKM	0.10	0.04	-0.18	0.28	0.18	-0.16	-0.05
	POU2F1	-0.09	0.00	0.03	-0.18	-0.20	0.07	0.10
	SCO2	-0.13	-0.25	-0.22	-0.11	-0.16	-0.14	-0.20
	STK11	-0.20	-0.27	-0.13	-0.17	0.19	-0.11	-0.26
	TP53	-0.02	-0.15	0.15	0.08	-0.16	-0.05	-0.02
FA Oxidation	ACAA1	-0.20	-0.27	-0.36	0.05	-0.33	-0.19	-0.16
	CPT1C	0.08	0.49	0.33	0.24	0.06	0.23	0.38
	CYP2E1	-0.03	0.06	-0.04	0.15	-0.10	0.03	0.02
	CYP4A11	-0.10	0.00	-0.02	0.07	-0.13	-0.09	0.05
	CYP4A22	-0.07	-0.09	-0.06	0.05	-0.13	-0.07	0.08
	PRKAA1	0.24	-0.05	0.16	0.01	-0.08	-0.13	0.04
Lipogenesis	ACACA	-0.10	-0.08	-0.17	0.03	-0.16	0.02	-0.12
	ACACB	0.11	0.29	-0.43	-0.02	0.02	0.18	0.40
	ACLY	-0.11	0.05	0.23	0.01	0.13	0.12	-0.15
	DGAT1	-0.18	-0.35	-0.22	-0.19	-0.34	-0.16	-0.20
	DGAT2	0.03	-0.02	-0.12	-0.09	-0.13	0.00	-0.08
	FASN	-0.12	-0.10	-0.15	-0.02	-0.13	0.03	-0.20
	LIPE	0.17	0.26	0.06	0.32	-0.01	0.14	0.28
	MGLL	0.09	-0.16	-0.01	-0.06	-0.19	-0.08	0.32
	MLXIPL	-0.08	-0.11	-0.08	-0.18	-0.06	-0.10	0.05
	NR1H3	0.02	0.17	0.00	-0.22	-0.05	-0.03	0.05
	NR1H4	0.07	-0.13	0.11	-0.02	-0.15	0.37	0.09
	NR1I2	0.05	-0.31	-0.08	0.06	-0.12	-0.06	0.21
	PPARA	0.25	-0.24	-0.23	0.14	-0.06	0.05	0.09
	PPARD	0.11	0.09	0.06	-0.04	0.06	0.09	0.30
	PPARG	0.20	-0.29	-0.46	0.27	-0.09	0.29	0.11
	RXRA	-0.09	-0.05	0.16	0.02	-0.09	0.11	0.08
	SREBF1	-0.29	-0.15	-0.18	-0.16	-0.25	-0.14	-0.33
FA Uptake	CAV1	0.55	0.62	0.37	0.39	0.29	0.39	0.58
	CD36	0.22	0.41	0.26	0.27	0.17	0.46	0.17
	FABP1	-0.02	-0.24	-0.12	-0.16	-0.09	-0.01	0.10
	FABP2	0.04	-0.22	-0.05	0.02	-0.09	0.07	0.14
	FABP3	-0.02	0.18	-0.36	0.30	-0.07	-0.03	0.26
	FABP4	0.25	0.35	0.03	0.34	0.26	0.35	0.09
	FABP5	0.13	-0.05	0.31	0.27	0.23	0.23	-0.12
	FABP6	-0.02	-0.18	-0.03	-0.06	-0.03	-0.10	-0.08
	FABP7	0.01	-0.06	0.11	0.20	0.06	0.29	0.00
	FABP9	-0.02	0.10	-0.08	0.08	-0.08	0.00	0.04
	PMP2	0.07	0.33	0.02	0.04	0.06	0.35	0.29
	SLC27A1	-0.15	0.24	-0.36	0.21	-0.15	-0.10	-0.24
	SLC27A2	-0.13	-0.34	0.00	0.00	-0.23	-0.05	-0.30
	SLC27A3	-0.22	0.08	0.19	-0.29	-0.13	-0.12	0.02
	SLC27A4	-0.18	-0.22	-0.47	-0.05	-0.08	-0.01	-0.38
	SLC27A5	-0.18	-0.16	-0.15	0.10	-0.17	-0.10	-0.17
	SLC27A6	0.08	0.19	-0.06	0.21	0.03	-0.04	0.15

Supplementary Figure 3: EMT score correlation heatmap

Heatmap indicating the Pearson's correlation coefficient of the different metabolic genes (Y-axis) with EMT score within a given cancer type (X-axis). Red indicates high magnitude of positive correlation, grey indicates negative correlation and white indicates low correlation. Horizontal blue boxes indicate patterns that are consistent across all cancer types, including positive correlation between *CAV1* and *CD36* with EMT scores, and negative correlation between *DGAT1* and *DGAT2* with EMT scores. Vertical blue boxes indicate patterns of a metabolic gene category consistent in a given cancer type, including negative correlation between genes in the FA oxidation and lipogenesis category with EMT score in lung cancer and negative correlation between Warburg effect genes and EMT score in ovarian cancer.

Suppl. Fig. 4

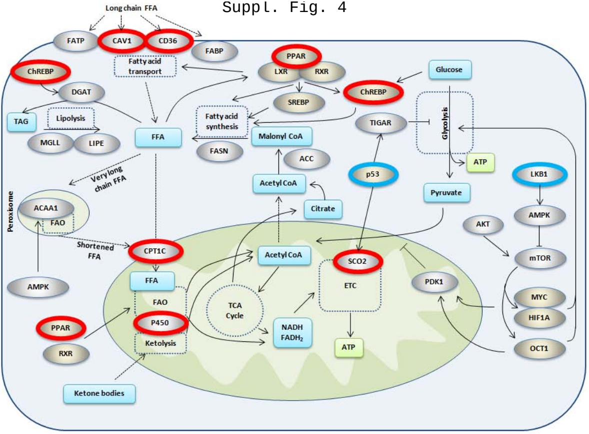

Long chain FFA
FATP
CAV1
CD36
FABP
Fatty acid transport
PPAR
LXR
RXR
Glucose
ChREBP
DGAT
SREBP
ChREBP
TAG
Lipolysis
Fatty acid synthesis
TIGAR
Glycolysis
MGLL
LIPE
FFA
Malonyl CoA
FASN
ACC
ATP
p53
LKB1
Very long chain FFA
Acetyl CoA
Pyruvate
Peroxisome
ACAA1
FAO
Citrate
AMPK
AKT
Shortened FFA
CPT1C
Acetyl CoA
SCO2
mTOR
FFA
ETC
PDK1
AMPK
FAO
TCA Cycle
MYC
PPAR
P450
NADH FADH2
HIF1A
Ketolysis
RXR
ATP
OCT1
Ketone bodies

Supplementary Figure 4: Summary of alterations in metastatic tumors

Metabolic pathway model modified to indicate the genes that were identified as significantly altered in metastatic tumors with significant impact on patient prognosis. The red-highlighted ovals show genes with metastasis-associated gain-of-function alterations with prognostic significance. The blue-highlighted ovals show the genes with loss-of-function alterations with prognostic significance in cancer but did not differ between primary and metastatic samples.

Suppl. Fig. 5

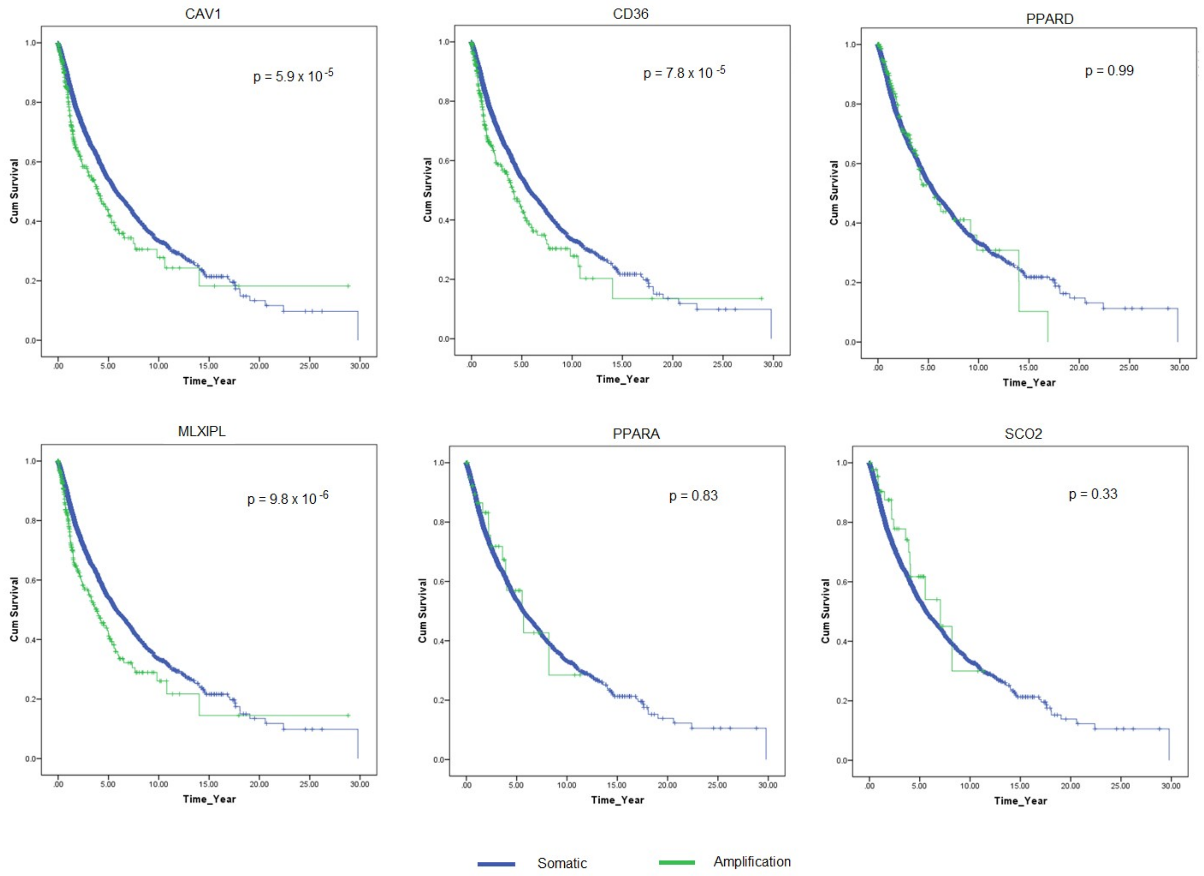

Supplementary Figure 5: Copy number and survival

Survival curves genes with significantly higher copy numbers in metastatic tumors in the pan-cancer dataset. P-values indicate significance levels from the comparison of survival curves using the log-rank (Mantel-Cox) test.

Supplementary Table 1: List of EMT score and metabolic model genes					
EMT: Mesenchymal	EMT: Epithelial	Warburg Effect	Fatty acid oxidation	Lipogenesis*	Fatty acid uptake
FN1	CLDN4	AKT1	PRKAA1	PPARA	SLC27A1
CDH2	CDH1	C12ORF5	ACAA1	ACACA	CAV1
SNAI1	CLDN7	HIF1A	CPT1C	ACACB	CD36
SNAI2	MUC1	MTOR	CYP2E1	ACLY	FABP1
TWIST1	TJP3	MYC	CYP4A11	DGAT1	FABP2
TWIST2		PDK1	CYP4A22	DGAT2	FABP3
VIM		PFKFB3		FASN	FABP4
ZEB1		PFKFB4		LIPE	FABP5
ZEB2		PKM		MGLL	FABP6
		POU2F1		MLXIPL	FABP7
		SCO2		NR1H3	FABP9
		STK11		NR1H4	PMP2
		TP53		NR1I2	SLC27A2
				PPARD	SLC27A3
				PPARG	SLC27A4
				RXRA	SLC27A5
				SREBF1	SLC27A6

* includes lipolysis and esterification genes

	Supplementary Table 2: Mutation frequency analysis (pancancer)					
	Gene	Primary Tumor mutation frequency	Metastatic tumor mutation frequency	Total mutation frequency	Fisher's Exact Test (2-tailed)	neg log10 (pvalue)
Warburg effect	C12orf5	0.232	0.897	0.264	0.171	0.767
	HIF1A	0.266	1.794	0.353	0.048	1.323
	MTOR	2.723	5.381	2.761	0.201	0.697
	MYC	0.498	1.794	0.558	0.060	1.222
	PDK1	0.365	0.897	0.382	0.271	0.567
	PFKFB3	0.365	0.448	0.353	1.000	0.000
	PFKFB4	0.299	1.794	0.382	0.027	1.561
	PKM2	0.399	1.345	0.441	0.135	0.871
	POU2F1	0.664	1.345	0.676	0.464	0.333
	SCO2	0.133	0.000	0.118	1.000	0.000
	STK11	0.996	1.345	0.969	0.440	0.356
FA oxidation	ACAA1	0.166	2.242	0.294	0.003	2.470
	CPT1C	0.764	4.036	0.940	0.002	2.699
	CYP2E1	0.465	4.933	0.734	0.000	5.562
	CYP4A11	0.797	9.417	1.322	0.000	11.671
	CYP4A22	0.664	6.278	1.028	0.000	7.674
	PRKAA1	0.266	0.448	0.264	1.000	0.000
Lipogenesis	ACACA	1.561	6.726	1.821	0.000	3.306
	ACACB	1.926	5.830	2.086	0.023	1.636
	ACLY	0.697	2.691	0.793	0.031	1.504
	DGAT1	0.266	0.897	0.294	0.210	0.677
	DGAT2	0.232	1.345	0.294	0.088	1.053
	FASN	0.930	6.726	1.263	0.000	6.274
	LIPE	0.664	3.587	0.823	0.004	2.452
	MGLL	0.166	2.691	0.323	0.000	4.556
	MLXIPL	0.598	4.933	0.852	0.000	4.986
	NR1H3	0.332	0.897	0.353	0.391	0.408
	NR1H4	0.764	4.933	0.999	0.000	4.588
	NR1I2	0.365	0.897	0.382	0.352	0.454
	PPARA	0.266	2.691	0.411	0.000	3.341
	PPARD	0.199	0.897	0.235	0.292	0.535
	PPARG	0.299	4.933	0.588	0.000	6.916
	RXRA	0.697	1.345	0.705	0.440	0.356
	SREBF1	0.631	2.242	0.705	0.026	1.584
FA uptake	CAV1	0.066	0.448	0.088	0.357	0.448
	CD36	0.232	1.794	0.323	0.027	1.561
	FABP1	0.199	2.691	0.353	0.000	3.621
	FABP2	0.100	2.242	0.235	0.001	3.244
	FABP3	0.100	0.000	0.088	1.000	0.000
	FABP4	0.100	0.448	0.118	0.357	0.448
	FABP5	0.100	0.000	0.088	1.000	0.000
	FABP6	0.166	0.000	0.147	1.000	0.000
	FABP7	0.100	0.000	0.088	1.000	0.000
	FABP9	0.199	0.897	0.235	0.114	0.941
	PMP2	0.100	1.345	0.176	0.029	1.535
	SLC27A1	0.365	1.345	0.411	0.187	0.727
	SLC27A2	0.697	4.933	0.940	0.000	4.882
	SLC27A3	0.232	1.345	0.294	0.078	1.107
	SLC27A4	0.465	1.345	0.499	0.187	0.727
	SLC27A5	0.498	2.691	0.617	0.007	2.139
	SLC27A6	0.864	9.417	1.410	0.000	10.927

Bonferroni adjusted p-value cutoff	9.80E-04

Category	Gene	Primary tumor gain frequency	Metastatic tumor gain frequency	Fisher's Exact Test (2-tailed)	neg log10 (pvalue)		Primary tumor loss frequency	Metastatic tumor loss frequency	Fisher's Exact Test (2-tailed)	neg log10 (pvalue)
Supplementary Table 3: Copy number frequency analysis (pancancer)										
Warburg effect	C12orf5	4.233	2.333	0.139	0.858		0.082	0.000	1.000	0.000
	HIF1A	0.737	0.000	0.278	0.557		0.070	0.333	0.215	0.669
	MTOR	0.608	1.000	0.435	0.361		0.117	0.000	1.000	0.000
	MYC	14.408	11.000	0.110	0.957					
	PDK1	0.924	0.000	0.116	0.936		0.035	0.000	1.000	0.000
	PFKFB3	2.234	0.000	0.002	2.691		0.409	1.000	0.137	0.864
	PFKFB4	0.363	0.000	0.626	0.203		0.105	0.000	1.000	0.000
	PKM2	0.713	1.667	0.072	1.140		0.023	0.000	1.000	0.000
	POU2F1	6.233	9.333	0.039	1.406		0.012	0.000	1.000	0.000
	SCO2	0.444	4.667	0.000	8.966		1.637	1.333	1.000	0.000
	STK11	0.573	1.333	0.104	0.982		0.433	0.000	0.637	0.196
	TP53	0.269	0.000	1.000	0.000		0.596	0.333	1.000	0.000
FA oxidation	ACAA1	0.491	0.333	1.000	0.000		0.035	0.000	1.000	0.000
	CPT1C	1.181	0.667	0.587	0.232		0.058	0.000	1.000	0.000
	CYP2E1	0.585	0.000	0.417	0.380		0.760	1.333	0.297	0.527
	CYP4A11	0.655	1.333	0.145	0.838		0.070	0.000	1.000	0.000
	CYP4A22	0.678	1.333	0.158	0.801		0.058	0.000	1.000	0.000
	PRKAA1	6.151	4.333	0.220	0.658					
Lipogenesis	ACACA	1.415	0.333	0.132	0.878		0.257	0.000	1.000	0.000
	ACACB	0.889	0.333	0.523	0.281		0.023	0.000	1.000	0.000
	ACLY	1.076	0.333	0.379	0.422		0.187	0.000	1.000	0.000
	DGAT1	10.899	10.667	1.000	0.000		0.082	0.000	1.000	0.000
	DGAT2	2.421	3.333	0.337	0.472		0.094	0.000	1.000	0.000
	FASN	2.269	2.000	1.000	0.000		0.094	0.333	0.267	0.574
	LIPE	1.555	0.667	0.332	0.478		0.117	0.000	1.000	0.000
	MGLL	3.017	1.333	0.115	0.938		0.023	0.000	1.000	0.000
	MLXIPL	3.462	8.000	0.000	3.644		0.035	0.000	1.000	0.000
	NR1H3	0.667	0.000	0.266	0.575		0.082	0.000	1.000	0.000
	NR1H4	0.912	0.000	0.116	0.937					
	NR1I2	2.924	1.000	0.050	1.298		0.035	0.000	1.000	0.000
	PPARA	0.444	5.000	0.000	9.908		1.216	0.333	0.270	0.569
	PPARD	1.579	10.333	0.000	14.398		0.047	0.000	1.000	0.000
	PPARG	1.041	0.000	0.077	1.115		0.047	0.000	1.000	0.000
	RXRA	0.760	0.000	0.174	0.759		0.164	0.667	0.101	0.997
	SREBF1	0.748	0.667	1.000	0.000		0.468	0.333	1.000	0.000
FA uptake	CAV1	3.812	8.333	0.000	3.388		0.012	0.000	1.000	0.000
	CD36	3.590	8.000	0.000	3.329		0.070	0.000	1.000	0.000
	FABP1	0.643	0.333	1.000	0.000					
	FABP2	0.444	0.000	0.640	0.194		0.187	0.000	1.000	0.000
	FABP3	0.550	1.333	0.094	1.029		0.058	0.000	1.000	0.000
	FABP4	8.233	10.000	0.286	0.544		0.047	0.000	1.000	0.000
	FABP5	8.245	10.333	0.201	0.697		0.047	0.000	1.000	0.000
	FABP6	1.029	0.667	0.771	0.113		0.187	0.333	0.444	0.353
	FABP7	0.772	0.333	0.729	0.137		0.105	1.000	0.007	2.170
	FABP9	8.221	10.000	0.285	0.545		0.047	0.000	1.000	0.000
	PMP2	8.233	10.000	0.286	0.544		0.047	0.000	1.000	0.000
	SLC27A1	2.234	0.667	0.069	1.161		0.012	0.000	1.000	0.000
	SLC27A2	0.363	0.667	0.309	0.511		0.211	0.667	0.146	0.836
	SLC27A3	7.262	9.667	0.115	0.938					
	SLC27A4	0.690	0.000	0.269	0.570		0.082	0.667	0.035	1.453
	SLC27A5	1.497	1.000	0.631	0.200		0.222	0.000	1.000	0.000
	SLC27A6	0.889	1.333	0.350	0.456		0.164	0.333	0.404	0.394

Bonferroni adjusted p-value cutoff: 9.62E-04

Supplementary Table 4: EMT score nearest neighbor analysis (BRCA)					
	Pearson's correlation	p-value	FDR (BH)	Bonferroni	FWER
TWIST1	0.393	0.002	0.003	0.136	0.000
ZEB2	0.679	0.002	0.003	0.136	0.000
ZEB1	0.593	0.002	0.003	0.136	0.000
FN1	0.492	0.002	0.003	0.136	0.000
SNAI2	0.591	0.002	0.003	0.136	0.000
VIM	0.633	0.002	0.003	0.136	0.000
TWIST2	0.486	0.002	0.003	0.136	0.000
CAV1	0.548	0.002	0.003	0.136	0.000
CD36	0.220	0.002	0.003	0.136	0.001
FABP4	0.249	0.002	0.003	0.136	0.000
LIPE	0.173	0.002	0.003	0.136	0.003
ACACB	0.110	0.004	0.006	0.271	0.073
PPARG	0.205	0.002	0.003	0.136	0.003
MGLL	0.092	0.002	0.003	0.136	0.238
MLXIPL	-0.076	0.016	0.021	1.000	0.610
FABP2	0.041	0.202	0.236	1.000	1.000
PFKFB3	0.039	0.255	0.294	1.000	1.000
CDH2	0.204	0.002	0.003	0.136	0.003
C12ORF5	0.273	0.002	0.003	0.136	0.000
PRKAA1	0.243	0.002	0.003	0.136	0.000
HIF1A	0.243	0.002	0.003	0.136	0.000
PPARA	0.253	0.002	0.003	0.136	0.000
PDK1	0.139	0.002	0.003	0.136	0.022
SNAI1	0.337	0.002	0.003	0.136	0.000
MYC	0.117	0.002	0.003	0.136	0.050
FABP5	0.128	0.002	0.003	0.136	0.033
PPARD	0.109	0.002	0.003	0.136	0.076
DGAT2	0.025	0.393	0.431	1.000	1.000
CPT1C	0.084	0.018	0.023	1.000	0.401
NR1I2	0.045	0.146	0.174	1.000	1.000
SLC27A6	0.082	0.014	0.019	0.950	0.461
TP53	-0.016	0.601	0.614	1.000	1.000
PMP2	0.074	0.032	0.039	1.000	0.652
FABP3	-0.016	0.605	0.614	1.000	1.000
CYP4A22	-0.070	0.022	0.028	1.000	0.751
POU2F1	-0.087	0.008	0.011	0.543	0.324
SLC27A2	-0.131	0.002	0.003	0.136	0.030
FABP9	-0.025	0.365	0.407	1.000	1.000
FABP7	0.012	0.615	0.615	1.000	1.000
FABP6	-0.021	0.493	0.516	1.000	1.000
PKM	0.098	0.004	0.006	0.271	0.162
PFKFB4	-0.112	0.002	0.003	0.136	0.065
AKT1	-0.185	0.002	0.003	0.136	0.003
MTOR	0.069	0.016	0.021	1.000	0.784
CDH1	-0.226	0.002	0.003	0.136	0.000
ACACA	-0.100	0.004	0.006	0.271	0.136
ACLY	-0.111	0.002	0.003	0.136	0.066
FASN	-0.117	0.002	0.003	0.136	0.052
SREBF1	-0.294	0.002	0.003	0.136	0.000
NR1H4	0.069	0.056	0.068	1.000	0.782
FABP1	-0.021	0.453	0.489	1.000	1.000
CYP2E1	-0.029	0.311	0.353	1.000	1.000
CYP4A11	-0.099	0.002	0.003	0.136	0.145
NR1H3	0.023	0.477	0.507	1.000	1.000
ACAA1	-0.196	0.002	0.003	0.136	0.003
SCO2	-0.132	0.002	0.003	0.136	0.028
SLC27A5	-0.176	0.002	0.003	0.136	0.003
DGAT1	-0.183	0.002	0.003	0.136	0.003
CLDN4	-0.368	0.002	0.003	0.136	0.000
CLDN7	-0.497	0.002	0.003	0.136	0.000
RXRA	-0.088	0.008	0.011	0.543	0.303
SLC27A1	-0.147	0.002	0.003	0.136	0.014
STK11	-0.197	0.002	0.003	0.136	0.003
TJP3	-0.555	0.002	0.003	0.136	0.000
MUC1	-0.419	0.002	0.003	0.136	0.000
SLC27A4	-0.177	0.002	0.003	0.136	0.003
SLC27A3	-0.217	0.002	0.003	0.136	0.001
EMT_SCORE	1.000	0.002	0.003	0.136	0.000

Significant genes (FWER)

Supplementary Table 5: EMT score nearest neighbor analysis (COADREAD)					
	Pearson's correlation	p-value	FDR (BH)	Bonferroni	FWER
VIM	0.882	0.002	0.003	0.136	0.000
ZEB2	0.848	0.002	0.003	0.136	0.000
TWIST2	0.788	0.002	0.003	0.136	0.000
ZEB1	0.735	0.002	0.003	0.136	0.000
CDH2	0.775	0.002	0.003	0.136	0.000
FN1	0.772	0.002	0.003	0.136	0.000
SNAI2	0.801	0.002	0.003	0.136	0.000
TWIST1	0.737	0.002	0.003	0.136	0.000
CAV1	0.619	0.002	0.003	0.136	0.000
SNAI1	0.631	0.002	0.003	0.136	0.000
HIF1A	0.437	0.002	0.003	0.136	0.000
PFKFB3	0.353	0.002	0.003	0.136	0.000
PMP2	0.334	0.002	0.003	0.136	0.000
CD36	0.407	0.002	0.003	0.136	0.000
FABP4	0.345	0.002	0.003	0.136	0.000
LIPE	0.255	0.002	0.003	0.136	0.004
ACACB	0.288	0.002	0.003	0.136	0.000
CPT1C	0.485	0.002	0.003	0.136	0.000
SLC27A6	0.194	0.022	0.032	1.000	0.059
FABP7	-0.065	0.178	0.212	1.000	1.000
FABP9	0.099	0.070	0.091	1.000	0.964
CYP2E1	0.064	0.200	0.234	1.000	1.000
PRKAA1	-0.050	0.347	0.385	1.000	1.000
CYP4A11	-0.001	0.916	0.930	1.000	1.000
CYP4A22	-0.094	0.044	0.061	1.000	0.983
MTOR	0.171	0.002	0.003	0.136	0.145
ACLY	0.046	0.371	0.401	1.000	1.000
FASN	-0.096	0.058	0.077	1.000	0.978
ACACA	-0.080	0.116	0.149	1.000	0.997
AKT1	0.044	0.405	0.431	1.000	1.000
PKM	0.036	0.495	0.518	1.000	1.000
SREBF1	-0.149	0.002	0.003	0.136	0.321
PDK1	-0.102	0.052	0.071	1.000	0.944
TP53	-0.149	0.002	0.003	0.136	0.326
POU2F1	-0.003	0.990	0.990	1.000	1.000
MLXIPL	-0.113	0.024	0.035	1.000	0.850
RXRA	-0.053	0.329	0.380	1.000	1.000
PPARA	-0.244	0.002	0.003	0.136	0.009
SLC27A1	0.243	0.002	0.003	0.136	0.009
SLC27A3	0.085	0.126	0.158	1.000	0.994
MUC1	-0.218	0.002	0.003	0.136	0.024
NR1H4	-0.135	0.002	0.003	0.136	0.525
PFKFB4	-0.111	0.042	0.059	1.000	0.875
FABP2	-0.217	0.002	0.003	0.136	0.026
PPARD	0.086	0.132	0.163	1.000	0.993
MGLL	-0.156	0.002	0.003	0.136	0.246
CLDN7	-0.486	0.002	0.003	0.136	0.000
TJP3	-0.515	0.002	0.003	0.136	0.000
CLDN4	-0.266	0.002	0.003	0.136	0.003
C12ORF5	-0.059	0.146	0.177	1.000	1.000
NR1H3	0.175	0.006	0.009	0.407	0.133
FABP3	0.183	0.006	0.009	0.407	0.096
DGAT2	-0.023	0.707	0.728	1.000	1.000
MYC	-0.047	0.351	0.385	1.000	1.000
FABP5	-0.053	0.349	0.385	1.000	1.000
SLC27A5	-0.158	0.002	0.003	0.136	0.233
SLC27A4	-0.217	0.002	0.003	0.136	0.026
SCO2	-0.246	0.002	0.003	0.136	0.009
DGAT1	-0.348	0.002	0.003	0.136	0.000
ACAA1	-0.272	0.002	0.003	0.136	0.001
STK11	-0.268	0.002	0.003	0.136	0.002
FABP6	-0.181	0.002	0.003	0.136	0.102
FABP1	-0.242	0.002	0.003	0.136	0.010
PPARG	-0.295	0.002	0.003	0.136	0.000
NR1I2	-0.310	0.002	0.003	0.136	0.000
CDH1	-0.343	0.002	0.003	0.136	0.000
SLC27A2	-0.343	0.002	0.003	0.136	0.000
EMT_SCORE	1.000	0.002	0.003	0.136	0.000

Significant genes (FWER)

Supplementary Table 6: EMT score nearest neighbor analysis (KIRC)					
	Pearson's correlation	p-value	FDR (BH)	Bonferroni	FWER
ZEB2	0.626	0.002	0.004	0.134	0.000
ZEB1	0.524	0.002	0.004	0.134	0.000
PRKAA1	0.161	0.002	0.004	0.134	0.141
VIM	0.486	0.002	0.004	0.134	0.000
CAV1	0.372	0.002	0.004	0.134	0.000
TP53	0.151	0.002	0.004	0.134	0.183
FN1	0.370	0.002	0.004	0.134	0.000
SNAI2	0.516	0.002	0.004	0.134	0.000
TWIST1	0.242	0.002	0.004	0.134	0.014
FABP5	0.314	0.002	0.004	0.134	0.000
SNAI1	0.385	0.002	0.004	0.134	0.000
CPT1C	0.335	0.002	0.004	0.134	0.000
AKT1	-0.073	0.076	0.106	1.000	0.985
C12ORF5	0.135	0.002	0.004	0.134	0.307
HIF1A	-0.005	0.922	0.951	1.000	1.000
MTOR	-0.018	0.685	0.740	1.000	1.000
ACACA	-0.172	0.004	0.007	0.267	0.098
PMP2	0.016	0.764	0.800	1.000	1.000
SLC27A6	-0.064	0.126	0.162	1.000	1.000
FABP7	0.113	0.006	0.010	0.401	0.533
CDH2	0.374	0.002	0.004	0.134	0.000
ACLY	0.229	0.002	0.004	0.134	0.019
PDK1	0.161	0.002	0.004	0.134	0.142
NR1H4	0.114	0.004	0.007	0.267	0.524
MYC	0.250	0.002	0.004	0.134	0.007
PFKFB3	0.214	0.002	0.004	0.134	0.027
PPARD	0.061	0.144	0.182	1.000	1.000
FABP2	-0.050	0.263	0.310	1.000	1.000
CD36	0.260	0.002	0.004	0.134	0.004
FABP4	0.035	0.425	0.491	1.000	1.000
LIPE	0.055	0.210	0.255	1.000	1.000
SLC27A2	0.003	0.946	0.960	1.000	1.000
CYP4A11	-0.017	0.707	0.751	1.000	1.000
CYP4A22	-0.059	0.226	0.270	1.000	1.000
MLXIPL	-0.082	0.088	0.120	1.000	0.949
PPARA	-0.226	0.002	0.004	0.134	0.021
FABP9	-0.080	0.120	0.157	1.000	0.956
RXRA	0.155	0.002	0.004	0.134	0.164
NR1H3	0.002	1.000	1.000	1.000	1.000
FABP6	-0.025	0.531	0.603	1.000	1.000
PFKFB4	0.097	0.032	0.049	1.000	0.779
MGLL	-0.015	0.649	0.713	1.000	1.000
PKM	-0.182	0.002	0.004	0.134	0.067
SLC27A3	0.192	0.002	0.004	0.134	0.043
POU2F1	0.029	0.547	0.611	1.000	1.000
STK11	-0.131	0.004	0.007	0.267	0.339
SCO2	-0.217	0.002	0.004	0.134	0.024
DGAT1	-0.218	0.002	0.004	0.134	0.024
SLC27A1	-0.359	0.002	0.004	0.134	0.000
CYP2E1	-0.037	0.204	0.253	1.000	1.000
NR1I2	-0.085	0.104	0.139	1.000	0.925
DGAT2	-0.118	0.052	0.076	1.000	0.468
FABP1	-0.122	0.054	0.077	1.000	0.411
SLC27A5	-0.154	0.040	0.059	1.000	0.165
ACAA1	-0.358	0.002	0.004	0.134	0.000
FABP3	-0.361	0.002	0.004	0.134	0.000
FASN	-0.153	0.020	0.031	1.000	0.168
SREBF1	-0.178	0.004	0.007	0.267	0.084
MUC1	-0.455	0.002	0.004	0.134	0.000
CLDN7	-0.677	0.002	0.004	0.134	0.000
CLDN4	-0.539	0.002	0.004	0.134	0.000
PPARG	-0.464	0.002	0.004	0.134	0.000
ACACB	-0.435	0.002	0.004	0.134	0.000
SLC27A4	-0.467	0.002	0.004	0.134	0.000
TJP3	-0.762	0.002	0.004	0.134	0.000
CDH1	-0.685	0.002	0.004	0.134	0.000
EMT_SCORE	1.000	0.002	0.004	0.134	0.000

Significant genes (FWER)

Supplementary Table 7: EMT score nearest neighbor analysis (LIHC)					
	Pearson's correlation	p-value	FDR (BH)	Bonferroni	FWER
TWIST2	0.383	0.004	0.019	0.271	0.040
TWIST1	0.492	0.002	0.010	0.136	0.017
FABP4	0.335	0.012	0.035	0.814	0.050
LIPE	0.320	0.016	0.040	1.000	0.056
FABP3	0.304	0.018	0.041	1.000	0.086
SLC27A1	0.207	0.022	0.047	1.000	0.382
PPARG	0.273	0.016	0.040	1.000	0.127
CD36	0.274	0.008	0.029	0.543	0.125
CPT1C	0.245	0.018	0.041	1.000	0.198
SNAI2	0.620	0.002	0.010	0.136	0.000
ZEB2	0.605	0.002	0.010	0.136	0.000
VIM	0.551	0.002	0.010	0.136	0.000
CAV1	0.394	0.002	0.010	0.136	0.035
PKM	0.282	0.006	0.024	0.407	0.109
HIF1A	0.199	0.018	0.041	1.000	0.430
C12ORF5	0.233	0.008	0.029	0.543	0.243
FABP5	0.274	0.016	0.040	1.000	0.125
SLC27A6	0.211	0.038	0.076	1.000	0.359
FABP7	0.204	0.036	0.074	1.000	0.404
PPARD	-0.041	0.573	0.683	1.000	1.000
MYC	0.126	0.080	0.136	1.000	0.976
TP53	0.082	0.255	0.378	1.000	1.000
SNAI1	0.265	0.006	0.024	0.407	0.139
FABP9	0.080	0.194	0.293	1.000	1.000
PMP2	0.043	0.459	0.573	1.000	1.000
AKT1	0.030	0.621	0.728	1.000	1.000
MGLL	-0.061	0.375	0.521	1.000	1.000
SREBF1	-0.162	0.014	0.040	0.950	0.757
PFKFB3	-0.112	0.078	0.136	1.000	0.994
CLDN4	-0.452	0.002	0.010	0.136	0.020
CDH1	-0.332	0.002	0.010	0.136	0.050
CLDN7	-0.504	0.002	0.010	0.136	0.015
SLC27A3	-0.293	0.002	0.010	0.136	0.100
TJP3	-0.477	0.002	0.010	0.136	0.018
CYP2E1	0.149	0.050	0.092	1.000	0.865
PDK1	0.120	0.066	0.118	1.000	0.989
FABP2	0.019	0.703	0.796	1.000	1.000
FN1	0.167	0.044	0.083	1.000	0.712
FABP6	-0.056	0.371	0.521	1.000	1.000
ZEB1	0.494	0.002	0.010	0.136	0.017
PRKAA1	0.010	0.860	0.877	1.000	1.000
CDH2	0.007	0.854	0.877	1.000	1.000
MUC1	-0.081	0.178	0.275	1.000	1.000
PFKFB4	0.051	0.415	0.556	1.000	1.000
MTOR	0.045	0.493	0.599	1.000	1.000
ACACA	0.025	0.699	0.796	1.000	1.000
ACLY	0.012	0.862	0.877	1.000	1.000
FASN	-0.023	0.749	0.821	1.000	1.000
SLC27A5	0.102	0.146	0.236	1.000	0.999
ACAA1	0.050	0.425	0.556	1.000	1.000
CYP4A11	0.070	0.285	0.413	1.000	1.000
CYP4A22	0.046	0.453	0.573	1.000	1.000
SLC27A2	0.002	0.908	0.908	1.000	1.000
PPARA	0.138	0.044	0.083	1.000	0.945
NR1I2	0.055	0.419	0.556	1.000	1.000
ACACB	-0.022	0.770	0.832	1.000	1.000
RXRA	0.024	0.737	0.821	1.000	1.000
MLXIPL	-0.178	0.010	0.032	0.679	0.593
NR1H4	-0.018	0.864	0.877	1.000	1.000
DGAT2	-0.090	0.174	0.275	1.000	1.000
SLC27A4	-0.050	0.463	0.573	1.000	1.000
FABP1	-0.160	0.020	0.044	1.000	0.768
SCO2	-0.107	0.122	0.202	1.000	0.998
DGAT1	-0.187	0.006	0.024	0.407	0.518
POU2F1	-0.175	0.012	0.035	0.814	0.615
STK11	-0.173	0.010	0.032	0.679	0.645
NR1H3	-0.225	0.002	0.010	0.136	0.274
EMT_SCORE	1.000	1.000	0.002	0.010	0.136

Significant genes (FWER)

Supplementary Table 8: EMT score nearest neighbor analysis (LUAD)					
	Pearson's correlation	p-value	FDR (BH)	Bonferroni	FWER
FN1	0.609	0.002	0.006	0.136	0.000
ZEB1	0.658	0.002	0.006	0.136	0.000
ZEB2	0.534	0.002	0.006	0.136	0.000
VIM	0.644	0.002	0.006	0.136	0.000
SNAI2	0.681	0.002	0.006	0.136	0.000
TWIST2	0.562	0.002	0.006	0.136	0.000
CDH2	0.508	0.002	0.006	0.136	0.000
TWIST1	0.525	0.002	0.006	0.136	0.000
PKM	0.180	0.026	0.043	1.000	0.451
SNAI1	0.467	0.002	0.006	0.136	0.000
HIF1A	0.464	0.002	0.006	0.136	0.000
C12ORF5	0.303	0.002	0.006	0.136	0.037
CAV1	0.289	0.006	0.015	0.407	0.044
FABP5	0.229	0.004	0.010	0.271	0.154
FABP4	0.265	0.004	0.010	0.271	0.071
CD36	0.174	0.024	0.042	1.000	0.506
PFKFB3	0.058	0.359	0.403	1.000	1.000
MLXIPL	-0.065	0.279	0.328	1.000	1.000
PFKFB4	0.150	0.044	0.068	1.000	0.761
STK11	0.187	0.008	0.019	0.543	0.389
PPARD	0.055	0.409	0.441	1.000	1.000
FABP6	-0.031	0.659	0.679	1.000	1.000
CDH1	-0.305	0.002	0.006	0.136	0.035
CLDN4	-0.405	0.002	0.006	0.136	0.000
FABP9	-0.083	0.104	0.138	1.000	1.000
FABP7	0.062	0.232	0.284	1.000	1.000
ACACB	0.017	0.760	0.772	1.000	1.000
PPARA	-0.056	0.403	0.441	1.000	1.000
ACLY	0.126	0.070	0.101	1.000	0.942
MTOR	0.095	0.160	0.209	1.000	1.000
ACACA	-0.158	0.016	0.033	1.000	0.679
SLC27A6	0.029	0.493	0.516	1.000	1.000
CPT1C	0.064	0.297	0.343	1.000	1.000
LIPE	-0.012	0.920	0.920	1.000	1.000
DGAT2	-0.132	0.022	0.042	1.000	0.915
CYP2E1	-0.105	0.086	0.119	1.000	0.998
PRKAA1	-0.082	0.194	0.244	1.000	1.000
PDK1	0.176	0.018	0.036	1.000	0.485
NR1H3	-0.051	0.415	0.441	1.000	1.000
FABP2	-0.088	0.094	0.128	1.000	1.000
FABP1	-0.092	0.060	0.089	1.000	1.000
NR1I2	-0.125	0.024	0.042	1.000	0.948
PPARG	-0.094	0.082	0.116	1.000	1.000
NR1H4	-0.148	0.012	0.028	0.814	0.779
MYC	0.145	0.038	0.060	1.000	0.808
TP53	-0.162	0.014	0.032	0.950	0.623
SLC27A2	-0.232	0.002	0.006	0.136	0.142
PMP2	0.055	0.361	0.403	1.000	1.000
FABP3	-0.068	0.234	0.284	1.000	1.000
SLC27A1	-0.151	0.024	0.042	1.000	0.759
MGLL	-0.192	0.002	0.006	0.136	0.354
SLC27A3	-0.131	0.026	0.043	1.000	0.918
CYP4A11	-0.128	0.030	0.048	1.000	0.935
CYP4A22	-0.133	0.024	0.042	1.000	0.908
SLC27A4	-0.078	0.259	0.310	1.000	1.000
RXRA	-0.087	0.176	0.225	1.000	1.000
FASN	-0.133	0.016	0.033	1.000	0.906
AKT1	-0.128	0.060	0.089	1.000	0.935
SREBF1	-0.246	0.002	0.006	0.136	0.108
POU2F1	-0.203	0.002	0.006	0.136	0.270
MUC1	-0.457	0.002	0.006	0.136	0.000
TJP3	-0.492	0.002	0.006	0.136	0.000
SCO2	-0.156	0.016	0.033	1.000	0.704
SLC27A5	-0.171	0.004	0.010	0.271	0.541
ACAA1	-0.332	0.002	0.006	0.136	0.016
DGAT1	-0.338	0.002	0.006	0.136	0.015
CLDN7	-0.505	0.002	0.006	0.136	0.000
EMT_SCORE	1.000	1.000	0.002	0.006	0.136

Significant genes (FWER)

Supplementary Table 9: EMT score nearest neighbor analysis (OV)					
	Pearson's correlation	p-value	FDR (BH)	Bonferroni	FWER
TWIST1	0.471	0.002	0.006	0.136	0.000
SNAI2	0.727	0.002	0.006	0.136	0.000
TWIST2	0.669	0.002	0.006	0.136	0.000
ZEB1	0.718	0.002	0.006	0.136	0.000
VIM	0.646	0.002	0.006	0.136	0.000
CAV1	0.394	0.002	0.006	0.136	0.000
ZEB2	0.491	0.002	0.006	0.136	0.000
NR1H4	0.375	0.002	0.006	0.136	0.002
PMP2	0.349	0.002	0.006	0.136	0.009
PPARG	0.291	0.006	0.016	0.407	0.030
FN1	0.381	0.002	0.006	0.136	0.001
SNAI1	0.489	0.002	0.006	0.136	0.000
CD36	0.463	0.002	0.006	0.136	0.000
FABP4	0.349	0.002	0.006	0.136	0.010
LIPE	0.137	0.044	0.093	1.000	0.803
FABP7	0.291	0.012	0.029	0.814	0.030
FABP5	0.228	0.004	0.012	0.271	0.116
PPARD	0.092	0.154	0.227	1.000	0.999
FABP2	0.069	0.248	0.343	1.000	1.000
PFKFB4	0.078	0.200	0.283	1.000	1.000
PDK1	0.035	0.495	0.612	1.000	1.000
ACACB	0.180	0.008	0.021	0.543	0.344
POU2F1	0.069	0.279	0.380	1.000	1.000
ACLY	0.115	0.074	0.132	1.000	0.959
ACACA	0.023	0.711	0.755	1.000	1.000
FASN	0.029	0.667	0.731	1.000	1.000
MTOR	-0.100	0.090	0.145	1.000	0.990
FABP1	-0.008	0.990	0.990	1.000	1.000
CPT1C	0.228	0.004	0.012	0.271	0.114
CYP2E1	0.031	0.567	0.688	1.000	1.000
NR1I2	-0.060	0.337	0.450	1.000	1.000
CYP4A22	-0.072	0.192	0.277	1.000	1.000
MLXIPL	-0.097	0.084	0.139	1.000	0.996
CDH2	0.313	0.002	0.006	0.136	0.022
SLC27A1	-0.102	0.046	0.095	1.000	0.990
TP53	-0.048	0.407	0.532	1.000	1.000
C12ORF5	0.028	0.635	0.708	1.000	1.000
FABP9	-0.003	0.910	0.938	1.000	1.000
SLC27A2	-0.046	0.463	0.583	1.000	1.000
RXRA	0.110	0.084	0.139	1.000	0.975
SLC27A4	-0.008	0.898	0.938	1.000	1.000
STK11	-0.114	0.064	0.117	1.000	0.963
AKT1	-0.082	0.140	0.211	1.000	1.000
DGAT2	-0.002	0.970	0.985	1.000	1.000
FABP6	-0.105	0.050	0.100	1.000	0.990
FABP3	-0.034	0.585	0.688	1.000	1.000
MYC	-0.097	0.106	0.167	1.000	0.994
SLC27A5	-0.099	0.078	0.136	1.000	0.992
HIF1A	0.036	0.587	0.688	1.000	1.000
PFKFB3	0.031	0.609	0.702	1.000	1.000
SLC27A6	-0.037	0.625	0.708	1.000	1.000
MGLL	-0.083	0.120	0.185	1.000	1.000
PPARA	0.047	0.431	0.553	1.000	1.000
CDH1	-0.314	0.002	0.006	0.136	0.022
NR1H3	-0.026	0.701	0.755	1.000	1.000
SLC27A3	-0.117	0.060	0.113	1.000	0.948
PRKAA1	-0.131	0.038	0.083	1.000	0.845
CLDN7	-0.381	0.002	0.006	0.136	0.001
CYP4A11	-0.094	0.056	0.109	1.000	0.998
SCO2	-0.137	0.020	0.047	1.000	0.805
SREBF1	-0.141	0.024	0.054	1.000	0.757
MUC1	-0.420	0.002	0.006	0.136	0.000
TJP3	-0.446	0.002	0.006	0.136	0.000
PKM	-0.162	0.010	0.025	0.679	0.513
DGAT1	-0.160	0.006	0.016	0.407	0.534
ACAA1	-0.195	0.002	0.006	0.136	0.236
CLDN4	-0.467	0.002	0.006	0.136	0.000
EMT_SCORE	1.000	1.000	0.002	0.006	0.136

Significant genes (FWER)

Supplementary Table 10: EMT score nearest neighbor analysis (PRAD)					
	Pearson's correlation	p-value	FDR (BH)	Bonferroni	FWER
TWIST2	0.428	0.002	0.005	0.136	0.000
VIM	0.639	0.002	0.005	0.136	0.000
ZEB1	0.712	0.002	0.005	0.136	0.000
ZEB2	0.598	0.002	0.005	0.136	0.000
FN1	0.534	0.002	0.005	0.136	0.000
PFKFB3	0.400	0.002	0.005	0.136	0.000
SNAI1	0.319	0.002	0.005	0.136	0.005
CAV1	0.575	0.002	0.005	0.136	0.000
SNAI2	0.436	0.002	0.005	0.136	0.000
ACACB	0.399	0.002	0.005	0.136	0.000
LIPE	0.275	0.002	0.005	0.136	0.032
CPT1C	0.378	0.002	0.005	0.136	0.000
NR1I2	0.211	0.004	0.008	0.271	0.195
PMP2	0.286	0.002	0.005	0.136	0.021
FABP4	0.092	0.154	0.218	1.000	1.000
C12ORF5	0.134	0.044	0.075	1.000	0.860
CDH2	0.332	0.002	0.005	0.136	0.002
MGLL	0.319	0.002	0.005	0.136	0.005
SLC27A6	0.151	0.034	0.059	1.000	0.687
PPARG	0.114	0.098	0.151	1.000	0.980
FABP9	0.043	0.397	0.482	1.000	1.000
TWIST1	0.323	0.002	0.005	0.136	0.005
FABP2	0.142	0.052	0.086	1.000	0.783
FABP1	0.098	0.138	0.199	1.000	0.999
POU2F1	0.099	0.100	0.151	1.000	0.997
PPARD	0.305	0.002	0.005	0.136	0.013
AKT1	-0.085	0.192	0.251	1.000	1.000
TP53	-0.024	0.717	0.761	1.000	1.000
NR1H4	0.087	0.188	0.250	1.000	1.000
RXRA	0.075	0.257	0.318	1.000	1.000
DGAT2	-0.083	0.204	0.261	1.000	1.000
CYP4A22	0.078	0.257	0.318	1.000	1.000
FABP7	-0.002	0.992	0.992	1.000	1.000
FABP6	-0.084	0.174	0.236	1.000	1.000
CYP4A11	0.046	0.455	0.523	1.000	1.000
CYP2E1	0.020	0.768	0.804	1.000	1.000
FABP3	0.264	0.002	0.005	0.136	0.043
MLXIPL	0.050	0.425	0.507	1.000	1.000
PKM	-0.050	0.441	0.517	1.000	1.000
SLC27A3	0.023	0.679	0.733	1.000	1.000
NR1H3	0.049	0.461	0.523	1.000	1.000
SLC27A5	-0.166	0.006	0.012	0.407	0.536
SCO2	-0.199	0.002	0.005	0.136	0.263
SLC27A1	-0.243	0.002	0.005	0.136	0.095
STK11	-0.255	0.002	0.005	0.136	0.061
ACAA1	-0.155	0.012	0.023	0.814	0.638
SLC27A4	-0.376	0.002	0.005	0.136	0.000
DGAT1	-0.197	0.006	0.012	0.407	0.281
CLDN7	-0.502	0.002	0.005	0.136	0.000
CLDN4	-0.321	0.002	0.005	0.136	0.005
TJP3	-0.377	0.002	0.005	0.136	0.000
MUC1	-0.430	0.002	0.005	0.136	0.000
HIF1A	0.102	0.128	0.189	1.000	0.995
PDK1	-0.033	0.559	0.613	1.000	1.000
MYC	0.012	0.824	0.837	1.000	1.000
PRKAA1	0.041	0.559	0.613	1.000	1.000
ACACA	-0.122	0.062	0.098	1.000	0.945
PPARA	0.094	0.164	0.227	1.000	1.000
MTOR	0.016	0.810	0.835	1.000	1.000
ACLY	-0.153	0.024	0.043	1.000	0.672
CDH1	-0.265	0.002	0.005	0.136	0.043
SLC27A2	-0.296	0.002	0.005	0.136	0.017
CD36	0.168	0.018	0.033	1.000	0.511
FABP5	-0.121	0.058	0.094	1.000	0.953
PFKFB4	-0.202	0.004	0.008	0.271	0.249
FASN	-0.202	0.008	0.016	0.543	0.248
SREBF1	-0.334	0.002	0.005	0.136	0.001
EMT_SCORE	1.000	1.000	0.002	0.005	0.136

Significant genes (FWER) (highlighted)

Supplementary Table 11: Logistic regression analysis (EMT score)

BRCA			COADREAD			KIRC			LIHC		
Gene	Odds Ratio	Neg. log10 (p-value)	Gene	Odds Ratio	Neg. log10 (p-value)	Gene	Odds Ratio	Neg. log10 (p-value)	Gene	Odds Ratio	Neg. log10 (p-value)
CAV1	12.161	24.933	CPT1C	302886	5.736	CPT1C	3.247	5.998	CPT1C	34.241	3.663
SLC27A3	0.744	2.600	NR1H4	0.122	4.005	RXRA	1.919	2.632	FABP5	7.805	2.971
C12ORF5	1.243	2.410	LIPE	0.037	3.090	FABP5	1.878	2.541	SREBF1	0.103	2.715
SLC27A1	0.718	2.246	CAV1	25.018	2.468	SCO2	0.516	2.505	CYP4A11	5.431	2.191
POU2F1	0.801	2.121	PPARA	0.219	2.411	MLXIPL	0.524	2.408	CYP2E1	3.350	2.062
CYP2E1	3.406	1.581	SLC27A2	4.644	2.313	FASN	0.560	2.200	SLC27A4	2.577	1.784
ACAA1	0.763	1.488	SCO2	0.197	2.036	CD36	2.098	1.846	ACLY	0.366	1.707
NR1H4	3.805	1.375	CYP2E1	1.831	2.031	PKM	0.609	1.727	CAV1	3.493	1.630
PKM	1.199	1.362	SLC27A3	0.304	1.886	CAV1	1.395	1.591	DGAT1	0.680	1.609
CPT1C	1.151	1.287	FABP4	497.701	1.796	STK11	0.620	1.527	ACACA	2.403	1.446
FABP1	0.982	1.238	FABP3	2.240	1.686	SLC27A5	0.468	1.388	PPARA	2.357	1.400
DGAT2	0.801	1.232	CYP4A22	2.271	1.563	LIPE	0.621	1.373	HIF1A	2.756	1.356
ACACB	0.770	1.194	PDK1	2.065	1.470	TP53	1.354	1.244	MLXIPL	0.480	1.351
PPARA	1.195	1.130	NR1H3	2.453	1.453	HIF1A	1.497	1.212	SLC27A6	1.798	1.049
SLC27A2	0.687	1.129	HIF1A	5.812	1.427	SREBF1	1.313	1.142	AKT1	1.548	1.026
AKT1	0.873	1.122	SLC27A1	2.421	1.420	MTOR	1.449	1.045	RXRA	0.557	0.980
CYP4A11	0.618	0.976	RXRA	2.672	1.392	ACAA1	1.550	0.978	PKM	0.265	0.960
FASN	1.152	0.938	SLC27A5	2.172	1.387	PFKFB4	1.433	0.974	MTOR	0.490	0.909
SREBF1	0.808	0.916	TP53	0.530	1.379	FABP1	0.688	0.894	FASN	2.213	0.871
PFKFB3	0.858	0.902	DGAT1	0.490	1.334	ACACB	0.788	0.846	PDK1	1.714	0.855
SCO2	1.187	0.825	FABP1	0.392	1.315	SLC27A3	1.298	0.789	NR1H3	0.512	0.842
DGAT1	0.931	0.690	ACACA	0.372	1.261	FABP3	0.770	0.765	MGLL	0.611	0.716
MLXIPL	0.858	0.634	PRKAA1	0.417	1.186	PPARA	1.398	0.758	DGAT2	1.531	0.670
FABP6	0.896	0.557	AKT1	0.546	1.117	ACACA	0.771	0.735	SLC27A1	0.414	0.643
PMP2	1.047	0.526	CD36	18.451	1.065	MYC	1.286	0.730	POU2F1	1.368	0.582
ACLY	0.927	0.511	NR1I2	2.090	1.035	SLC27A4	1.269	0.721	FABP7	0.124	0.571
NR1I2	0.942	0.491	PFKFB3	0.378	1.020	DGAT2	1.271	0.702	CYP4A22	0.537	0.550
PDK1	1.096	0.438	SLC27A4	0.396	0.998	FABP4	0.630	0.665	PRKAA1	1.386	0.514
PPARG	0.873	0.389	PPARD	2.088	0.945	FABP2	1.190	0.572	SLC27A2	0.698	0.448
SLC27A5	1.073	0.381	MGLL	0.458	0.877	PPARG	0.766	0.562	NR1H4	1.289	0.370
CD36	0.922	0.365	FABP2	0.301	0.864	CYP2E1	1.426	0.541	ACAA1	0.663	0.355
FABP5	1.020	0.358	FABP9	0.001	0.773	SLC27A2	0.797	0.497	FABP1	1.422	0.354
TP53	0.948	0.357	MTOR	1.702	0.669	FABP7	1.107	0.447	PFKFB3	0.768	0.348
MTOR	0.948	0.277	ACLY	1.910	0.601	CYP4A11	1.279	0.327	FABP2	0.712	0.321
FABP3	0.938	0.245	FABP6	1.485	0.460	CYP4A22	0.827	0.309	PPARG	0.559	0.304
MYC	0.958	0.222	SLC27A6	2.078	0.335	NR1H3	1.114	0.297	PFKFB4	0.778	0.251
MGLL	1.045	0.203	PPARG	1.373	0.272	DGAT1	1.135	0.244	SLC27A3	0.872	0.251
NR1H3	1.051	0.192	ACAA1	1.198	0.249	C12ORF5	1.079	0.233	PMP2	1.102	0.235
PFKFB4	0.955	0.182	ACACB	1.317	0.220	MGLL	0.918	0.215	C12ORF5	1.182	0.230
HIF1A	1.041	0.179	STK11	1.230	0.218	NR1H4	0.948	0.157	FABP4	0.554	0.206
FABP9	0.992	0.158	FABP7	1.274	0.216	PRKAA1	0.931	0.134	ACACB	1.256	0.199
FABP2	0.964	0.153	PKM	1.230	0.186	SLC27A1	0.923	0.129	FABP6	0.844	0.176
SLC27A4	1.039	0.151	MLXIPL	1.100	0.179	PFKFB3	1.060	0.122	MYC	0.878	0.164
FABP7	0.989	0.150	PFKFB4	0.826	0.136	NR1I2	1.023	0.105	FABP9	2.395	0.162
STK11	1.042	0.129	PMP2	1.180	0.120	POU2F1	1.047	0.103	PPARD	0.866	0.158
LIPE	1.068	0.111	FASN	1.152	0.086	PMP2	1.033	0.087	LIPE	4.884	0.139
RXRA	1.016	0.063	FABP5	0.935	0.080	PDK1	1.023	0.052	CD36	0.875	0.117
PRKAA1	0.993	0.030	POU2F1	0.949	0.053	PPARD	1.022	0.051	SLC27A5	1.178	0.115
ACACA	0.995	0.028	CYP4A11	0.947	0.053	ACLY	1.016	0.027	NR1I2	0.898	0.094
FABP4	0.982	0.027	SREBF1	1.078	0.051	FABP9	1.016	0.015	SCO2	1.052	0.048
SLC27A6	1.005	0.024	DGAT2	1.050	0.044	AKT1	1.008	0.015	TP53	0.962	0.035
PPARD	0.996	0.019	MYC	0.985	0.022	FABP6	0.997	0.007	STK11	1.027	0.033
CYP4A22	0.996	0.011	C12ORF5	1.005	0.011	SLC27A6	1.001	0.004	FABP3	0.848	0.013

LUAD			OV			PRAD			Cumulative rank	
Gene	Odds Ratio	Neg. log10 (p-value)	Gene	Odds Ratio	Neg. log10 (p-value)	Gene	Odds Ratio	Neg. log10 (p-value)	Gene	Rank sum
HIF1A	18.125	4.394	CD36	14.305	3.484	CAV1	42.335	3.903	CPT1C	286
ACACB	2.364	2.578	NR1H4	20.058	2.184	NR1I2	6.795	3.019	CAV1	255
PFKFB4	0.146	2.080	CPT1C	2.270	2.132	CPT1C	12.796	2.877	CD36	223
PDK1	2.584	1.877	SLC27A3	0.541	2.127	CD36	6.493	2.795	MLXIPL	213
RXRA	2.674	1.778	PFKFB4	1.887	1.886	SLC27A1	0.066	2.773	CYP2E1	209
PPARD	1.965	1.496	FASN	1.300	1.182	FABP5	0.348	1.874	SLC27A3	208
TP53	0.403	1.355	MTOR	0.672	1.141	TP53	0.359	1.779	RXRA	197
PMP2	1.743	1.218	PRKAA1	0.785	1.106	DGAT1	4.245	1.742	PFKFB4	193
AKT1	0.582	1.176	SLC27A6	1.347	0.902	POU2F1	3.175	1.603	PPARA	193
STK11	2.252	1.154	FABP2	0.581	0.869	MGLL	2.941	1.413	AKT1	189
FABP2	0.096	1.130	PPARD	1.237	0.821	FABP4	25.773	1.157	ACACB	188
CD36	1.426	0.947	NR1H3	1.413	0.787	PFKFB4	0.408	1.145	NR1H4	188
POU2F1	0.557	0.929	MLXIPL	0.661	0.740	NR1H3	4.397	1.142	HIF1A	186
DGAT2	0.631	0.913	MYC	1.222	0.711	LIPE	0.214	1.085	MTOR	185
NR1I2	0.423	0.873	FABP9	0.008	0.669	SCO2	3.983	1.083	FASN	180
FABP7	1.6E+14	0.806	AKT1	0.907	0.646	FABP3	0.204	0.971	LIPE	180
MTOR	0.615	0.772	SLC27A5	0.832	0.619	ACACA	2.281	0.924	PKM	180
MYC	0.565	0.691	LIPE	0.726	0.593	PMP2	0.585	0.877	FABP1	177
MLXIPL	0.247	0.679	PMP2	0.524	0.548	SREBF1	0.439	0.845	SCO2	176
C12ORF5	1.425	0.640	FABP3	0.871	0.444	FABP2	60.629	0.813	PDK1	175
FABP4	0.662	0.569	FABP1	1.137	0.423	MLXIPL	0.435	0.791	SREBF1	175
CYP2E1	0.745	0.526	DGAT2	0.868	0.410	PRKAA1	2.060	0.748	TP53	174
CYP4A11	1.580	0.521	FABP4	1.978	0.406	ACACB	2.224	0.714	FABP2	173
FABP3	1.333	0.464	ACACB	1.175	0.394	PDK1	0.465	0.695	DGAT1	171
SLC27A2	0.690	0.434	CYP4A11	0.909	0.391	FABP1	10.593	0.661	SLC27A1	171
FABP9	1.294	0.427	SLC27A4	1.268	0.374	STK11	0.325	0.593	NR1H3	170
NR1H4	0.391	0.385	SLC27A2	1.202	0.350	C12ORF5	1.526	0.538	FABP3	169
CAV1	1.181	0.384	PKM	0.866	0.344	ACAA1	0.431	0.533	DGAT2	167
SCO2	0.717	0.373	ACAA1	0.863	0.325	RXRA	0.651	0.522	FABP4	167
SLC27A5	0.620	0.361	PPARA	1.196	0.325	AKT1	1.699	0.513	FABP5	167
LIPE	1.246	0.333	DGAT1	0.953	0.276	PKM	1.722	0.487	SLC27A2	165
FABP6	1.300	0.330	SREBF1	0.835	0.268	PPARA	1.811	0.479	POU2F1	162
SLC27A3	0.731	0.326	ACLY	1.171	0.246	FABP6	0.398	0.474	ACAA1	161
CYP4A22	0.651	0.319	CYP2E1	1.186	0.217	DGAT2	0.699	0.454	CYP4A11	154
PKM	0.776	0.290	FABP6	1.089	0.207	CYP2E1	0.724	0.412	PMP2	151
SLC27A1	0.716	0.271	PPARG	1.104	0.196	SLC27A3	0.609	0.345	SLC27A5	149
SREBF1	0.744	0.268	RXRA	0.889	0.195	FASN	1.545	0.330	ACACA	146
FASN	0.733	0.259	C12ORF5	0.945	0.184	SLC27A6	1.256	0.313	NR1I2	137
ACAA1	0.689	0.253	PDK1	0.956	0.139	MYC	1.344	0.300	PRKAA1	133
MGLL	0.775	0.236	HIF1A	1.059	0.138	MTOR	0.749	0.279	C12ORF5	132
ACACA	0.818	0.223	ACACA	0.914	0.136	ACLY	0.668	0.265	MGLL	125
PPARA	0.854	0.217	FABP5	1.063	0.131	HIF1A	0.687	0.257	MYC	123
PPARG	0.668	0.202	POU2F1	0.950	0.120	PPARD	1.382	0.219	SLC27A4	123
FABP1	0.000	0.160	CAV1	1.089	0.120	CYP4A11	1.308	0.207	PPARD	116
CPT1C	1.103	0.149	CYP4A22	1.086	0.117	FABP9	1.433	0.189	STK11	114
NR1H3	1.083	0.109	TP53	0.942	0.107	FABP7	0.510	0.166	ACLY	110
PRKAA1	0.933	0.090	MGLL	1.071	0.098	SLC27A5	0.683	0.163	FABP6	97
PFKFB3	0.927	0.080	SCO2	0.958	0.072	PFKFB3	1.232	0.147	FABP9	97
SLC27A4	0.943	0.064	PFKFB3	1.026	0.046	SLC27A4	1.127	0.066	SLC27A6	96
ACLY	1.048	0.063	NR1I2	1.013	0.025	CYP4A22	0.926	0.065	CYP4A22	94
FABP5	0.938	0.061	FABP7	1.042	0.023	SLC27A2	1.073	0.059	FABP7	93
DGAT1	0.974	0.035	SLC27A1	0.992	0.022	PPARG	1.051	0.040	PPARG	88
SLC27A6	0.981	0.031	STK11	0.997	0.004	NR1H4	1.008	0.010	PFKFB3	82

Supplementary Table 12: GSEA analysis (oncogene signature) with CAV1+CD36 expression clusters (pancan)

NAME	NOM p-val	FDR q-val	FWER p-val
HOXA9_DN.V1_UP	0.000	0.007	0.005
STK33_SKM_UP	0.000	0.013	0.040
RAF_UP.V1_UP	0.000	0.015	0.054
STK33_UP	0.000	0.016	0.050
STK33_NOMO_UP	0.000	0.019	0.037
EGFR_UP.V1_UP	0.000	0.025	0.037
TBK1.DF_UP	0.000	0.045	0.221
GCNP_SHH_UP_EARLY.V1_DN	0.000	0.048	0.258
ATF2_UP.V1_DN	0.002	0.025	0.104
P53_DN.V2_DN	0.002	0.067	0.440
E2F1_UP.V1_DN	0.002	0.024	0.110
PDGF_UP.V1_DN	0.002	0.037	0.169
RPS14_DN.V1_UP	0.002	0.013	0.015
WNT_UP.V1_DN	0.002	0.046	0.259
CAHOY_ASTROGLIAL	0.002	0.016	0.040
RB_DN.V1_DN	0.004	0.042	0.197
CRX_DN.V1_DN	0.004	0.046	0.272
CSR_LATE_UP.V1_DN	0.006	0.045	0.241
PKCA_DN.V1_DN	0.006	0.050	0.313
KRAS.DF.V1_UP	0.006	0.055	0.357
NOTCH_DN.V1_DN	0.006	0.053	0.333
LTE2_UP.V1_UP	0.008	0.051	0.296
AKT_UP.V1_DN	0.008	0.047	0.270
P53_DN.V1_DN	0.008	0.017	0.064
ESC_J1_UP_LATE.V1_UP	0.010	0.029	0.140
PRC2_SUZ12_UP.V1_DN	0.010	0.077	0.490
TGFB_UP.V1_DN	0.010	0.078	0.490
CORDENONSI_YAP_CONSERVED_SIGNATURE	0.010	0.027	0.125
RELA_DN.V1_DN	0.013	0.066	0.448
ATM_DN.V1_UP	0.014	0.111	0.683
PTEN_DN.V2_UP	0.016	0.066	0.443
ATF2_S_UP.V1_DN	0.018	0.056	0.367
PRC2_EDD_UP.V1_DN	0.019	0.083	0.525
STK33_SKM_DN	0.019	0.116	0.703
PRC2_EZH2_UP.V1_DN	0.019	0.069	0.428
RB_P130_DN.V1_DN	0.020	0.064	0.409
JNK_DN.V1_UP	0.021	0.079	0.546
ESC_V6.5_UP_EARLY.V1_DN	0.021	0.044	0.226
KRAS.PROSTATE_UP.V1_UP	0.022	0.082	0.543
ATM_DN.V1_DN	0.023	0.080	0.546
PIGF_UP.V1_UP	0.024	0.050	0.302
CYCLIN_D1_UP.V1_DN	0.025	0.139	0.777
IL15_UP.V1_DN	0.028	0.109	0.666
JNK_DN.V1_DN	0.030	0.110	0.686
LTE2_UP.V1_DN	0.031	0.068	0.434
TGFB_UP.V1_UP	0.032	0.057	0.355
RELA_DN.V1_UP	0.033	0.087	0.583
CYCLIN_D1_KE_.V1_DN	0.034	0.125	0.724
ALK_DN.V1_UP	0.036	0.082	0.539
EIF4E_DN	0.037	0.076	0.493
KRAS.600_UP.V1_DN	0.041	0.103	0.630
LEF1_UP.V1_UP	0.045	0.078	0.485
CRX_DN.V1_UP	0.046	0.144	0.792
NOTCH_DN.V1_UP	0.050	0.166	0.873
CTIP_DN.V1_UP	0.050	0.137	0.779
BCAT_BILD_ET_AL_DN	0.053	0.083	0.534
NRL_DN.V1_DN	0.055	0.169	0.869
ESC_V6.5_UP_LATE.V1_UP	0.056	0.110	0.676
KRAS.600.LUNG.BREAST_UP.V1_UP	0.057	0.124	0.724
KRAS.600.LUNG.BREAST_UP.V1_DN	0.058	0.161	0.832
KRAS.LUNG_UP.V1_UP	0.060	0.134	0.757
PKCA_DN.V1_UP	0.066	0.147	0.800
BMI1_DN.V1_UP	0.069	0.085	0.534
STK33_NOMO_DN	0.071	0.168	0.849
BRCA1_DN.V1_DN	0.073	0.190	0.913
SNF5_DN.V1_DN	0.074	0.133	0.747
IL21_UP.V1_DN	0.075	0.167	0.871
BCAT_GDS748_UP	0.076	0.160	0.829
KRAS.LUNG.BREAST_UP.V1_DN	0.079	0.163	0.839
MEK_UP.V1_DN	0.079	0.133	0.758
BMI1_DN_MEL18_DN.V1_UP	0.081	0.101	0.631
KRAS.LUNG.BREAST_UP.V1_UP	0.081	0.133	0.750
AKT_UP_MTOR_DN.V1_DN	0.086	0.187	0.901
IL2_UP.V1_UP	0.086	0.139	0.779
RAF_UP.V1_DN	0.087	0.110	0.683
IL2_UP.V1_DN	0.092	0.173	0.887
PDGF_ERK_DN.V1_UP	0.093	0.205	0.944
MEL18_DN.V1_UP	0.098	0.106	0.649
KRAS.BREAST_UP.V1_UP	0.100	0.169	0.849
P53_DN.V2_UP	0.104	0.167	0.856
MEK_UP.V1_UP	0.105	0.136	0.780
ERB2_UP.V1_UP	0.106	0.167	0.851
PTEN_DN.V1_UP	0.108	0.168	0.870
BCAT.100_UP.V1_DN	0.110	0.190	0.903
KRAS.BREAST_UP.V1_DN	0.110	0.191	0.915
PTEN_DN.V1_DN	0.112	0.190	0.918
GLI1_UP.V1_UP	0.117	0.186	0.898
SNF5_DN.V1_UP	0.117	0.168	0.862
ESC_J1_UP_EARLY.V1_UP	0.118	0.207	0.943
YAP1_UP	0.125	0.203	0.934
KRAS.AMP.LUNG_UP.V1_UP	0.128	0.190	0.914
BRCA1_DN.V1_UP	0.132	0.193	0.922
PRC1_BMI_UP.V1_DN	0.133	0.203	0.937
GCNP_SHH_UP_LATE.V1_DN	0.137	0.203	0.939
ESC_V6.5_UP_LATE.V1_DN	0.140	0.211	0.948
KRAS.KIDNEY_UP.V1_DN	0.141	0.197	0.930
KRAS.PROSTATE_UP.V1_DN	0.142	0.202	0.937
CTIP_DN.V1_DN	0.142	0.218	0.952
KRAS.50_UP.V1_DN	0.145	0.161	0.835
STK33_DN	0.149	0.231	0.964
PDGF_UP.V1_UP	0.153	0.230	0.962
CSR_EARLY_UP.V1_DN	0.155	0.200	0.933
KRAS.300_UP.V1_DN	0.155	0.192	0.913
AKT_UP.V1_UP	0.156	0.192	0.910
RAPA_EARLY_UP.V1_UP	0.156	0.217	0.951
MEL18_DN.V1_DN	0.161	0.190	0.913
DCA_UP.V1_UP	0.167	0.241	0.966
MYC_UP.V1_DN	0.169	0.192	0.913
CAMP_UP.V1_DN	0.177	0.203	0.934
IL21_UP.V1_UP	0.179	0.228	0.960
LEF1_UP.V1_DN	0.183	0.191	0.917
MTOR_UP.N4.V1_DN	0.191	0.191	0.923
VEGF_A_UP.V1_DN	0.204	0.206	0.943
NFE2L2.V2	0.207	0.249	0.972
ESC_J1_UP_EARLY.V1_DN	0.209	0.245	0.969
IL15_UP.V1_UP	0.210	0.229	0.962
BCAT.100_UP.V1_UP	0.210	0.221	0.955
RB_P107_DN.V1_DN	0.211	0.240	0.966
JAK2_DN.V1_UP	0.217	0.251	0.972
TBK1.DF_DN	0.217	0.209	0.948
MTOR_UP.V1_DN	0.222	0.250	0.970
CRX_NRL_DN.V1_DN	0.223	0.251	0.972
CYCLIN_D1_UP.V1_UP	0.232	0.267	0.975
KRAS.LUNG_UP.V1_DN	0.245	0.227	0.961
VEGF_A_UP.V1_UP	0.267	0.272	0.975
MTOR_UP.N4.V1_UP	0.270	0.289	0.981
AKT_UP_MTOR_DN.V1_UP	0.274	0.268	0.975
EGFR_UP.V1_DN	0.275	0.299	0.986
BMI1_DN.V1_DN	0.289	0.291	0.982
KRAS.50_UP.V1_UP	0.294	0.250	0.972
SRC_UP.V1_DN	0.311	0.337	0.989
PRC2_SUZ12_UP.V1_UP	0.316	0.341	0.992
BMI1_DN_MEL18_DN.V1_DN	0.320	0.313	0.986
CYCLIN_D1_KE_.V1_UP	0.320	0.329	0.988
YAP1_DN	0.325	0.339	0.992
KRAS.600_UP.V1_UP	0.336	0.331	0.988
ERB2_UP.V1_DN	0.369	0.333	0.989
P53_DN.V1_UP	0.385	0.339	0.992
NRL_DN.V1_UP	0.390	0.396	0.995
CSR_EARLY_UP.V1_UP	0.416	0.392	0.995
SIRNA_EIF4GI_DN	0.423	0.399	0.997
KRAS.DF.V1_DN	0.434	0.422	0.997
CRX_NRL_DN.V1_UP	0.445	0.428	0.997
KRAS.300_UP.V1_UP	0.464	0.450	0.997
HOXA9_DN.V1_DN	0.492	0.476	0.997
GLI1_UP.V1_DN	0.582	0.546	0.999
KRAS.AMP.LUNG_UP.V1_DN	0.608	0.580	0.999
SINGH_KRAS_DEPENDENCY_SIGNATURE_	0.636	0.694	1.000
TBK1.DN.48HRS_DN	0.646	0.619	0.999
PRC1_BMI_UP.V1_UP	0.687	0.603	0.999
BCAT_BILD_ET_AL_UP	0.693	0.727	1.000
MTOR_UP.V1_UP	0.696	0.693	1.000
GCNP_SHH_UP_LATE.V1_UP	0.706	0.753	1.000
RAPA_EARLY_UP.V1_DN	0.724	0.624	0.999
DCA_UP.V1_DN	0.725	0.620	0.999
E2F3_UP.V1_DN	0.780	0.712	1.000
SIRNA_EIF4GI_UP	0.790	0.726	1.000
ATF2_S_UP.V1_UP	0.923	0.770	1.000
BCAT_GDS748_DN	0.965	0.937	1.000

	CD36+CAV1 expression clusters	
	High	Low
Number of significant gene sets (P <0.05, FDR <25%)	54	1
Percentage of significant oncogenic signatures	28.88	0.53
Number of gene sets (oncogenic signatures)	187	
